# A 14-gene B-cell immune signature in early-stage triple-negative breast cancer (TNBC): a pooled analysis of seven studies

**DOI:** 10.1016/j.ebiom.2024.105043

**Published:** 2024-03-05

**Authors:** Benedetta Conte, Fara Brasó-Maristany, Adela Rodríguez Hernández, Tomás Pascual, Guillermo Villacampa, Francesco Schettini, Maria J. Vidal Losada, Elia Seguí, Laura Angelats, Isabel Garcia-Fructuoso, Raquel Gómez-Bravo, Natàlia Lorman-Carbó, Laia Paré, Mercedes Marín-Aguilera, Olga Martínez-Sáez, Barbara Adamo, Esther Sanfeliu, Beatrice Fratini, Claudette Falato, Núria Chic, Ana Vivancos, Patricia Villagrasa, Johan Staaf, Joel S. Parker, Charles M. Perou, Aleix Prat

**Affiliations:** a Translational Genomics and Targeted Therapies in Solid Tumors Group, August Pi i Sunyer Biomedical Research Institute (IDIBAPS), Barcelona, Spain; b Cancer Institute and Blood Diseases, Hospital Clinic de Barcelona, Barcelona, Spain; c Reveal Genomics, Barcelona, Spain; d SOLTI Cooperative Group, Barcelona, Spain; e Oncology Data Science, Vall d'Hebron Institute of Oncology, Barcelona, Spain; f Department of Medicine, University of Barcelona, Barcelona, Spain; g Institute of Oncology (IOB)-Hospital QuirónSalud, Barcelona, Spain; h Pathology Department, Hospital Clinic of Barcelona, Barcelona, Spain; i Vall d’Hebron Institute of Oncology (VHIO), Cancer Genomics Group, Barcelona, Spain; j Division of Translational Cancer Research, Department of Laboratory Medicine, Lund University, Sweden; k Department of Genetics, University of North Carolina, Chapel Hill, NC, USA; l Lineberger Comprehensive Cancer Center, University of North Carolina, Chapel Hill, NC, USA

**Keywords:** Triple-negative breast cancer (TNBC), Pathological complete response (pCR), Event-free survival (EFS), Overall survival (OS), B-cell/immunoglobulin signature (IGG), Prognostic biomarkers, Predictive biomarkers, Gene expression

## Abstract

**Background:**

Early-stage triple-negative breast cancer (TNBC) displays clinical and biological diversity. From a biological standpoint, immune infiltration plays a crucial role in TNBC prognosis. Currently, there is a lack of genomic tools aiding in treatment decisions for TNBC. This study aims to assess the effectiveness of a B-cell/immunoglobulin signature (IGG) alone, or in combination with tumor burden, in predicting prognosis and treatment response in patients with TNBC.

**Methods:**

Genomic and clinical data were retrieved from 7 cohorts: SCAN-B (N = 874), BrighTNess (n = 482), CALGB-40603 (n = 389), METABRIC (n = 267), TCGA (n = 118), GSE58812 (n = 107), GSE21653 (n = 67). IGG and a risk score integrating IGG with tumor/nodal staging (IGG-Clin) were assessed for event-free survival (EFS) and overall survival (OS) in each cohort. Random effects model was used to derive pooled effect sizes. Association of IGG with pathological complete response (pCR) was assessed in CALGB-40603 and BrighTNess. Immune significance of IGG was estimated through CIBERSORTx and EcoTyper.

**Findings:**

IGG was associated with improved EFS (pooled HR = 0.77, [95% CI = 0.70–0.85], I^2^ = 18%) and OS (pooled HR = 0.79, [0.73–0.85], I^2^ = 0%) across cohorts, and was predictive of pCR in CALGB-40603 (OR 1.25, [1.10–1.50]) and BrighTNess (OR 1.57 [1.25–1.98]). IGG-Clin was predictive of recurrence (pooled HR = 2.11, [1.75–2.55], I^2^ = 0%) and death (pooled HR = 1.99, 95% [0.84–4.73], I^2^ = 79%) across cohorts. IGG was associated with adaptive immune response at CIBERSORTx and EcoTyper analysis.

**Interpretation:**

IGG is linked to improved prognosis and pCR in early-stage TNBC. The integration of IGG alongside tumor and nodal staging holds promise as an approach to identify patients benefitting from intensified or de-intensified treatments.

**Funding:**

This study received funding from: Associació Beca Marta Santamaria, European Union’s Horizon 2020 research and innovation and Marie Skłodowska–Curie Actions programs, Fundación FERO, Fundación CRIS contra el cáncer, Agència de Gestó d'Ajuts Universitaris i de Recerca, 10.13039/501100004587Instituto de Salud Carlos III, Fundación Contigo, Asociación Cáncer de Mama Metastásico IV, 10.13039/100001006Breast Cancer Research Foundation, RESCUER, Fundación científica AECC and FSEOM.

**Research in context**

**Evidence before this study**

Neoadjuvant chemotherapy with anthracyclines and taxanes has long been the standard treatment for most patients with early-stage TNBC. However, recent advancements have introduced escalated and de-escalated strategies to tailor treatment intensity based on prognosis. For instance, anthracyclines-free regimens combining carboplatin and a taxane have recently emerged as a less toxic treatment option in the neoadjuvant setting. On the other hand, escalated approaches, such as the incorporation of immune checkpoint inhibitors and platinum agents into anthracyclines-containing regimens, have shown to reduce the risk of recurrence in this context. Currently, decisions whether to endorse patients for such approaches are still based on tumor size and nodal status in the pre-treatment context, and on the evaluation of tumor response in the post-neoadjuvant setting. Nevertheless, there is a growing recognition of the substantial role played by tumor biology, particularly its immunological features, in determining patient outcomes. Consequently, there is a pressing need for tools that integrate the prognostic information from these features into a single assay to guide systemic therapy effectively.

**Added value of this study**

This study represents an attempt to construct a prognostic score for early TNBC by combining clinical and genomic immune variables. We have identified a 14-gene immunoglobulin B-cell signature (IGG) as an independent predictor of pCR and survival outcomes in patients with early-stage TNBC. The IGG signature provided significant prognostic information beyond tumor size and nodal status, and it predicted distinct survival outcomes in patients with low tumor burden. Building upon this discovery, we evaluated the IGG-Clin risk-score, based on the HER2DX genomic assay for HER2+ breast cancer, which comprehensively assesses both the expression of the IGG signature and tumor stage. The IGG-Clin risk-score successfully identified patients at significantly different risks of relapse and death across cohorts.

**Implications of all the available evidence**

Integrating the prognostic information obtained from both IGG expression and tumor stage into a unified assay holds the potential to enhance treatment decision-making in early-stage TNBC. This approach could prove particularly beneficial for patients whose tumors fall into low-risk groups, as it may enable the use of less aggressive treatment strategies while still achieving curative outcomes. Further studies will be crucial to establish the clinical utility of integrating immune-genomic and clinical features into a prognostic assay to personalize treatment for patients with TNBC.

## Introduction

Early-stage triple-negative breast cancer (TNBC) has a poor prognosis despite (neo)adjuvant multi-agent chemotherapy.^1^^,^^2^ Classical factors associated with risk of recurrence are tumor size and nodal status.^3^ These two clinical-pathological factors dictate the possibility to combine multi-agent chemotherapy with pembrolizumab, an immune checkpoint inhibitor (ICI).^4^ However, TNBC is clinically and biologically heterogeneous, and immune-related features can influence prognosis beyond type of treatment and tumor burden.^5^, ^6^, ^7^, ^8^, ^9^, ^10^, ^11^, ^12^, ^13^, ^14^, ^15^, ^16^ Therefore, a standardized assay integrating tumor burden with immune-related characteristics could improve prognostic stratification and treatment personalization.

In the last decade, the abundance of tumoral immune infiltrate has emerged as a prominent molecular biomarker in TNBC.^7^^,^^10^^,^^17^ Tumor-infiltrating lymphocytes (TILs) have been associated with pathological complete response (pCR) in patients receiving neoadjuvant chemotherapy,^9^^,^^10^ and with improved survival in patients treated with and without chemotherapy.^7^^,^^12^ Among the diverse TILs subpopulations, the presence of CD8+ T-lymphocytes, assessed either with immunohistochemistry or gene expression, has shown a strong prognostic impact,^18^, ^19^, ^20^, ^21^ while the role of B-lymphocytes has long been overlooked. Recently, compelling evidence has suggested that B-lymphocytes could indeed play a pivotal role in TNBC outcomes, particularly when they exhibit an antigen-driven phenotype.^22^^,^^23^ For instance, the oligoclonal expansion of type G immunoglobulins (IgG) in the tumor stroma of patients with early-stage TNBC was strongly associated with long-term survival in the CALGB-40603 neoadjuvant trial.^24^

Similarly, a 14-gene immunoglobulin B-cell signature (IGG) has been identified as a key variable associated with a better prognosis and a higher probability of pCR in HER2-positive (HER2+) breast cancer treated with anti-HER2-based chemotherapy.^25^, ^26^, ^27^, ^28^ The IGG signature in combination with tumor burden (i.e., tumor size and nodal status), and two other gene signatures tracking proliferation and luminal features, have been integrated into a single assay, called HER2DX, and validated retrospectively across >7 studies and >1800 patients. This genomic test is currently available for clinical use.^25^^,^^29^^,^^30^

Here, we aimed to clinically validate the association of the IGG signature with prognosis and/or pCR across various cohorts of patients with TNBC. In addition, we aimed to assess the prognostic value of a multi-feature assay based on the HER2DX prognostic algorithm when IGG, tumor size and nodal status are considered.

## Methods

### Patient datasets

**Fig. 1:**
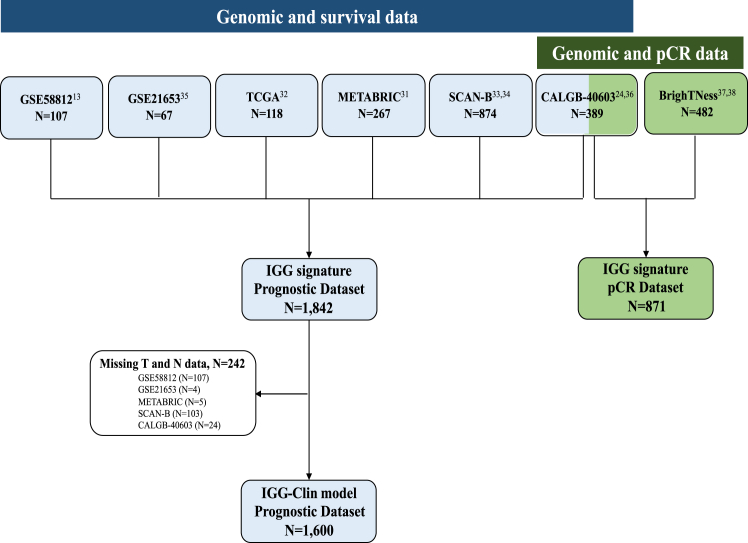
**Overview of the various patient cohorts assessed**.

A summary of all the cohorts evaluated is available in Table S1 and Fig. 1. Genomic, clinical and survival data were extracted from 5 non-overlapping, publicly available datasets of patients with TNBC. Data from The Cancer Genome Atlas (TCGA) and METABRIC were retrieved from the cBio Cancer Genomics Portal (http://cbioportal.org).^31^^,^^32^ Data from the last updated version of the SCAN-B dataset was retrieved from Mendeley Data (https://data.mendeley.com/datasets/yzxtxn4nmd).^33^^,^^34^ Two other publicly available datasets were obtained from the NCBI Gene Expression Omnibus (GEO) repository (https://www.ncbi.nlm.nih.gov) under the accession numbers GSE21653, and GSE58812.^13^^,^^35^ Patients were eligible for inclusion if they exhibited early-stage breast tumors classified as triple-negative by immunohistochemistry and had available gene expression data along with either disease-related survival or overall survival data. In cases where the percentage of tumor nuclei staining positively for estrogen and progesterone receptors was accessible, tumors were deemed triple-negative if both receptor types were below 10%. For datasets that presented estrogen and progesterone receptor expression as binary variables (i.e., “positive” or “negative”), tumors were classified as triple-negative based on the specific definition provided within each dataset (Table S1). Patients with missing information of (neo)adjuvant treatments or who did not receive any were still regarded as eligible. Both females and male patients were eligible.

To further assess IGG and IGG-Clin in a more homogeneous and contemporary treatment setting and explore potential associations with pCR, two additional datasets of patients treated with neoadjuvant chemotherapy (NACT) were explored. The CALGB-40603 phase II trial randomized 443 patients with stage II-III TNBC to receive either standard NACT (12 cycles of weekly paclitaxel followed by 4 cycles of doxorubicin plus cyclophosphamide), standard NACT plus carboplatin, standard NACT plus bevacizumab, or standard NACT plus carboplatin and bevacizumab.^24^^,^^36^ Clinical data and RNA-seq data of pre-treatment baseline samples from 389 (88%) patients from CALGB-40603 were downloaded from the dbGaP web site, under accession number phs001863.v1.p1. The BrighTNess phase III, placebo-controlled trial randomized 634 patients with stage II-III TNBC to receive either standard NACT (same scheme as CALGB-40603), standard NACT plus carboplatin, or standard NACT plus carboplatin plus veliparib.^37^^,^^38^ Clinical data and RNA-seq data of pre-treatment baseline samples from 482 (76%) patients from BrighTNess were downloaded from the GEO repository web site, under accession number GSE164458. Survival data along with matched genomic data were not publicly available for the BrighTNess dataset. Direct download links to gene expression datasets are provided in the Supplementary methods.

### Gene expression

Gene expression was assessed with different genomic platforms across datasets (Table S1). Overall, there was moderate correlation between platforms, with the exception of dataset GSE58812 (Supplementary methods). Z-scores or log2-transformed normalized expression from either RNA-seq or Microarray data was used for single genes and to derive the IGG signature, depending on the type of data available for each dataset. Briefly, IGG has been previously identified by an unsupervised clustering of 550 node-negative breast tumors,^39^ and externally validated across several clinical cohorts, for a total of >1000 patients.^25^, ^26^, ^27^, ^28^^,^^40^ The IGG signature is composed of 14 genes implicated in maturation of T and B lymphocytes progenitors (*IL2RG*), CD4+ and B lymphocytes activation and survival (*CD27*, *TNFRSF17*, *PIM2*), B lymphocytes differentiation in germinal centers (*POU2AF1*), immunoglobulin production (*CD79a*, *JCHAIN*, *IGKC*, *IGL*, *IGLV3-25*), chemotaxis (*CXCL8*, *NTN3*), and regulation of B, T and NK lymphocytes activity (*LAX1*, *HLA-C*). In datasets where the expression of some IGG genes was missing, the signature score was calculated from the available genes (Figure S1). For consistency with previous studies, the signature was calculated as mean expression of genes.

### IGG-Clin score

IGG-Clin is based on the prognostic HER2DX algorithm developed for early-stage HER2+ breast cancer.^25^, ^26^, ^27^, ^28^^,^^41^, ^42^ Briefly, HER2DX risk-score is a clinical genomic test, which integrates clinical-pathological information (i.e., tumor size and nodal status) with the expression of 4 gene signatures, including IGG. The 2 clinical-pathological and the 4 genomic variables are multiplied by a risk coefficient to obtain a final HER2DX risk-score (from 0 to 100). In HER2DX, tumor size and nodal status are categorized as T1 vs T2-4 and N0 vs N1 vs N2-3, respectively. In addition, gene signatures are computed as continuous variables. IGG-Clin score is based on the same exact HER2DX risk-score algorithm without the luminal and proliferation signatures yet retains the risk coefficients for tumor size, nodal status and IGG score.

### Biological relevance of IGG signature

To evaluate the immune cell-type composition captured by the IGG signature, CIBERSORTx (https://cibersortx.stanford.edu/) and EcoTyper (https://ecotyper.stanford.edu/carcinoma/) publicly available tools were interrogated separately in each dataset.^43^^,^^44^ Differences in immune cell-type composition between IGG groups (i.e., quartiles), were compared using a 2-way ANOVA considering both IGG quartiles and datasets, followed by Tukey's honest significant difference analysis.

To further investigate the relevance of IGG in TNBC, we interrogated a larger panel of 185 breast cancer-related genes encompassing the 14 IGG genes, the 50 genes from the PAM50 subtypes,^45^^,^^46^ and 121 genes implicated in proliferation, luminal differentiation, HER2-signalling, and immune response. To assess whether the IGG genes would be selected for their prognostic and predictive relevance from this core panel, we examined the association of each gene with event-free survival (EFS) in the CALGB-40603 and SCAN-B datasets, and with pCR in the CALGB-40603 and BrighTNess datasets. Genes showing consistent association with both EFS and pCR across datasets were selected as core significant genes. Next, we aimed at demonstrating that the combined evaluation of core significant genes tracking a specific biological module, like the IGG signature, enhances the accuracy of EFS and pCR prediction compared to individual genes. To do so, we compared the goodness-of-fit of models combining just two core immune genes (corresponding to a minimal signature) to models using individual core immune genes. Finally, the goodness-of-fit of different methods of signatures calculation (i.e., addition, subtraction, multiplication and ratio) were also evaluated.

The univariate and multivariable Cox regression models and logistic regression models tested for the goodness-of-fit were adjusted for treatment arm. The goodness-of-fit of each model was measured by the chi-square (χ2) statistic obtained from a likelihood ratio test.

### Statistical analysis

All survival analyses were based on time-to-event data, with the start time set as the date of cancer diagnosis. For surrogate survival endpoints, the end time was defined according to the occurrence of the dataset-specific event of interest or at the loss of follow-up. Because surrogate survival endpoint definitions varied across datasets (Table S1), we uniformly adopted the term EFS in our reporting to represent these various endpoints. For the analysis of overall survival (OS), the end time was determined by death from any cause or the loss of follow-up. The association of IGG and IGG-Clin with disease-related survival outcomes was assessed separately for each dataset (Table S1) using uni- and multi-variable Cox model analyses. Variables known to be associated with survival in early breast cancer, i.e., age at diagnosis, tumor size (T1 vs T2-4), and nodal status (N0 vs N+), were included in the multivariable model.^3^^,^^47^ IGG and IGG-Clin were evaluated as continuous variables and as quartiles. Hazard ratios (HRs) from uni- and multi-variable analyses were pooled using random effects model according the Hartung-Knapp-Sidik-Jonkman method.^48^ To test the contribution of the IGG score, we estimated the log likelihood ratio statistic of IGG quartiles as an addition to a Cox model containing tumor size and nodal status. The definition of disease-related survival outcomes in each dataset can be found in Table S1. Of note, GSE58812 dataset was not included in the pooled analysis since no data on tumor size and nodal status was available.

The association of IGG signature with pCR following neoadjuvant chemotherapy (NACT) was assessed in the CALGB-40603^24^^,^^36^ and BrighTNess^37^^,^^38^^,^^49^ datasets using uni- and multi-variable logistic regression analyses to calculate the odds ratio (OR) and 95% Confidence Intervals (CIs). Variables known to be associated with pCR, i.e., tumor size (T1 vs T2-4), nodal status (N0 vs N+), and carboplatin receipt, were included in the multivariable model.^50^^,^^51^ IGG was evaluated as a continuous variable and as quartiles. Interaction tests were used to evaluate whether the benefit of carboplatin in pCR was similar between IGG subgroups. To test the interaction, a new covariate with the interaction between carboplatin and IGG status was included in the logistic model. pCR was defined as absence of invasive disease in breast and axilla (ypT0/Tis ypN0).

For all statistical analyses, the significance level was set to a 2-sided alpha of 0.05.

### Role of the funding source

The funding sources had no role in the study design, data interpretation, manuscript preparation and final submission.

### Ethics

The study was performed in accordance with Good Clinical Practice guidelines and the Declaration of Helsinki. No informed consents were needed for the present study. Data were retrieved form public repositories.

## Results

### Description of prognostic datasets

Survival follow-up was available for 1842 patients from SCAN-B, CALGB-40603, METABRIC, TCGA, GSE58812, and GSE21653 (Fig. 1). Datasets were substantially heterogeneous in terms of genomic data, clinical data, and follow-up (Table 1 and Tables S1 and S2). Microarray gene expression data was available for METABRIC, GSE58812 and GSE21653, while RNA-seq data was available for SCAN-B, CALGB-40603 and TCGA. Different surrogate survival endpoints were reported in each dataset: disease-free survival (DFS) in TCGA and GSE21653, distant-DFS in GSE58812, relapse-free interval (RFI) in SCAN-B, event-free survival (EFS) in CALGB-40603, and breast cancer specific survival (BCSS) in METABRIC. OS was missing in GSE21653 and CALGB-40603 datasets. Thereafter, we used the term EFS and OS across all datasets.

**Table 1:** Characteristics of patients in the prognostic datasets.

	TCGA	METABRIC	GSE21653	GSE58812	SCAN-B	CALGB 40,603	Total	Eligible for IGG-Clin
**N**	118	267	87	107	874	389	1842	1600
**Tumour size, N (%)**								
T1	30 (25.4)	104 (39.0)	16 (18.4)	–	454 (52.0)	42 (10.8)	646 (35.1)	634 (39.6)
T2-4	88 (74.6)	160 (60.0)	68 (78.2)	–	341 (39.0)	340 (87.4)	997 (54.1)	966 (60.4)
NA	0 (0)	3 (1.0)	3 (3.4)	107 (100)	79 (9.0)	7 (1.8)	199 (10.8)	0 (0)
**Nodal status, N (%)**								
N0	75 (63.6)	128 (47.9)	51 (58.6)	–	517 (59.2)	164 (42.1)	935 (50.8)	692 (43.3)
N+	43 (36.4)	139 (52.1)	34 (39.1)	–	289 (33.0)	201 (51.7)	706 (38.3)	908 (56.7)
NA	0 (0)	0 (0)	2 (2.3)	107 (100)	68 (7.8)	24 (6.2)	201 (10.9)	0 (0)
**Treatment, N (%)**								
None	15 (12.7)	113 (42.3)	–	–	207 (23.7)	0 (0)	335 (18.2)	323 (20.2)
(Neo)adjuvant CT	64 (54.2)	154 (57.7)	–	–	621 (71.0)	389 (100)	1228 (66.8)	1139 (71.2)
NA	39 (33.1)	0 (0)	87 (100)	107 (100)	46 (5.3)	0 (0)	279 (15.1)	138 (8.6)
**Median follow-up**, **years (IQR)**	2.8 (1.4–5.0)	14.4 (8.0–17)	4.8 (3.3–8.5)	7.0 (4.3–9.2)	5.0 (4.5–8.0)	5.7 (4.8–6.4)	6	6
**Disease specific events**	19	109	27	31	131	115	432	373
**Deaths**	16	142	NA	29	244	NA	431	376

NA, not available; CT, chemotherapy.

With respect to patients’ characteristics, 100% of them were females, 67% (n = 1228) received (neo)adjuvant chemotherapy, while all patients in GSE21653 (n = 67) and GSE58812 (n = 107), and 33% (n = 39) of patients in TCGA, had missing information regarding the use of systemic treatments. Of 1842 patients with survival follow-up, 1600 (86.9%) had IGG-Clin score available. In GSE58812 dataset (n = 107, 9.8% of the combined cohort), tumor size and nodal status were missing (Table 1, and Fig. 1).

### Association of IGG with EFS and OS

IGG as continuous variable was significantly associated with better EFS in SCAN-B (HR = 0.72, 95% CI = 0.62–0.83, p < 0.0001), CALGB-40603 (HR = 0.82, 95% CI = 0.68–0.99, p = 0.040), METABRIC (HR = 0.81, 95% CI = 0.67–0.99, p = 0.040), and GSE21653 (HR = 0.54, 95% CI = 0.35–0.82, p = 0.004). No significant association of IGG with EFS was observed in TCGA (HR = 0.86, 95% CI = 0.52–1.45, p = 0.577) and GSE58812 (HR = 1.00, 95% CI = 0.69–1.45, p = 0.778). With respect to OS, a significant association was observed in SCAN-B (HR = 0.78, 95% CI = 0.71–0.85, p < 0.0001) and METABRIC (HR = 0.81, 95% CI = 0.67–0.96, p = 0.041), while no association was observed in TCGA (HR = 0.63, 95% CI = 0.32–1.26, p = 0.319) and GSE58812 (HR = 0.99, 95% CI = 0.68–1.46, p = 0.723).

When results were pooled with a random effects model, IGG was found significantly associated with better EFS (HR = 0.77, 95% CI = 0.70–0.85, p = 0.003, heterogeneity statistic I^2^ = 18%) and better OS (HR = 0.79, 95% CI = 0.73–0.85, p = 0.002, I^2^ = 0%). The association of IGG with survival outcome was found to be independent of age (continuous variable), tumor size (i.e., T1 vs T2-4), nodal status (i.e., N0 vs N+), and receipt of (neo)adjuvant chemotherapy (i.e., no chemotherapy vs chemotherapy vs unknown) both for EFS (HR = 0.77, 95% CI = 0.70–0.84, p < 0.0001, heterogeneity statistic I^2^ = 11%) and OS (HR = 0.80, 95% CI = 0.73–0.88, p < 0.0001, heterogeneity statistic I^2^ = 0%) (Figures S2 and S3).

### IGG in node-negative and stage 1 disease

**Fig. 2:**
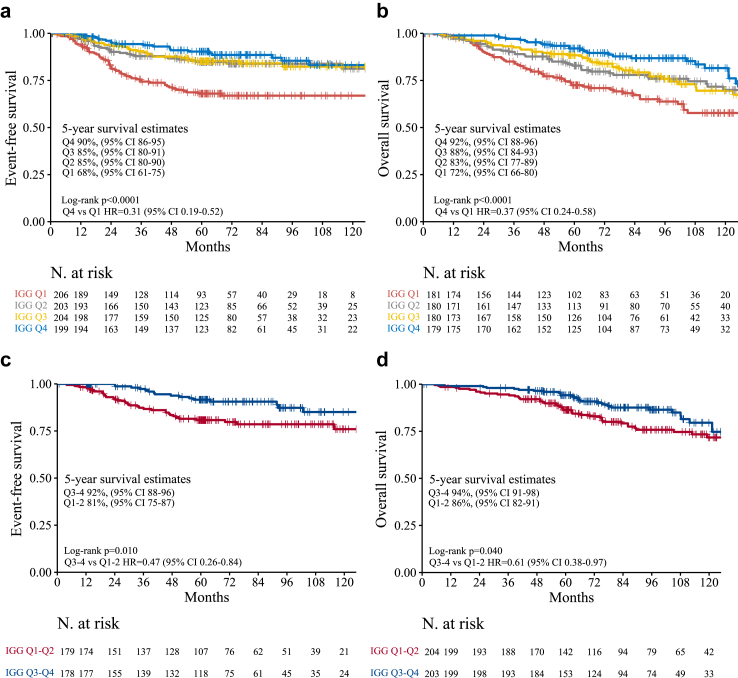
**Kaplan–Meier estimates in node-negative or stage 1 TNBC according to the expression of the IGG signature (quartiles).** (a) EFS in patients with node-negative disease; (b) OS in patients with node-negative disease; (c) EFS in patients with stage I disease; (d) OS in patients with stage I disease.

To identify the optimal patient population with TNBC for therapy de-escalation, those with node-negative or stage 1 disease were considered. To concentrate on these specific groups, each dataset was divided into four equal parts (quartiles) based on IGG expression levels. Subsequently, all datasets were merged to analyse survival associations across these quartiles. After a median follow-up period of 6.2 years, the quartiles of IGG expression demonstrated significant prognostic value in node-negative disease (n = 935) and stage 1 disease (n = 432), both in terms of EFS and OS (Fig. 2A–D). In node-negative disease, patients in the highest quartile showed a 69% and 63% relative reduction in the risk of event or death compared to those in the lowest quartile (EFS HR = 0.31, 95% CI = 0.19–0.52], p < 0.0001; OS HR = 0.37, 95% CI = 0.24–0.58, p = 0.001), which corresponded to a 5-year EFS and OS events rate of 10% and 8% in the highest quartile group, and of 32–28% in the lowest quartile group, respectively. In stage 1 disease, patients in the top two quartiles (i.e., IGG expression above median) showed a 54% and 39% relative reduction in the risk of event or death compared to those in the two lowest quartiles (i.e., below median) (EFS HR = 0.46, 95% CI = 0.25–0.84, p = 0.012; OS HR = 0.61, 95% CI = 0.38–0.97, p = 0.040). Such difference translated in to a 5-year EFS and OS event rate of 8% and 6% in the top two quartiles group and of 19% and 12% in the two lowest quartiles group, respectively (Fig. 2C and D).

### Prognostic value of IGG beyond tumor stage

**Fig. 3:**
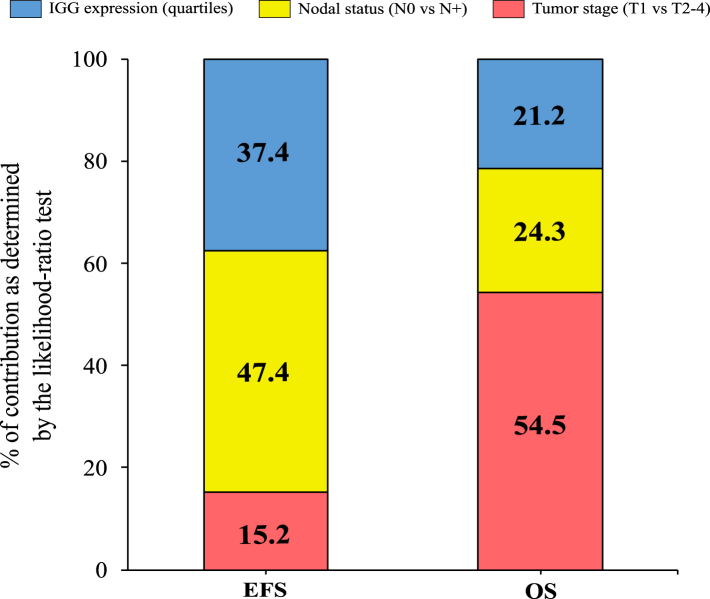
**Impact of the IGG signature beyond tumor stage.** Visual depiction showcasing the distinct contributions of variables to improve the overall fit of the prognostic model for both event-free survival (EFS) and overall survival (OS) outcomes. The sequence of variable inclusion in each model followed the order of tumor stage, nodal stage, and IGG signature. The assessment employed the likelihood-ratio chi-squared statistic as a measure of how well the model fits the data.

To evaluate the potential of a risk-score integrating IGG with tumor stage, we used the combined dataset (n = 1600) to build a multivariable Cox model containing (in sequential order) tumor stage, nodal status and IGG quartiles. Next, we estimated the relative contribution of each variable to the final log likelihood ratio statistic of the model, as a measure of the amount of prognostic information conveyed by each one these variables. The analysis demonstrated a substantial prognostic contribution of IGG expression beyond tumor stage, amounting to 37% for EFS and 21% for OS (Fig. 3).

### Association of IGG-Clin with EFS and OS

We assessed the IGG-Clin score, a comprehensive risk-score that combines IGG, tumor size, and nodal status into a unified measure. To create IGG-Clin, we used the risk coefficients of the previously reported HER2DX prognostic algorithm,^25^^,^^41^ which integrates tumor size, nodal status and IGG to obtain a continuous risk score which is positively associated with the risk of recurrence. Of note, we omitted the luminal and proliferation signatures which are also part of the HER2DX risk-score.

IGG-Clin risk score as a continuous variable was found significantly associated with both EFS and OS across all datasets (Table S3). Upon pooling the results from each dataset using a random effects model a significant effect size was observed for EFS (HR = 2.11, 95% CIs = 1.75–2.55, p = 0.0004, I^2^ = 0%), while the effect size for OS was not significant due to high heterogeneity (HR = 1.99, 95% CIs = 0.84–4.73, p = 0.076, I^2^ = 79%) (Figure S4A-B).

To further investigate the prognostic potential of IGG-Clin risk score, patients from each dataset were categorized into quartiles based on their IGG-Clin risk scores. The resulting data were combined, resulting in a pooled dataset of 1600 patients. Most patients (n = 966; 60%) had primary tumors larger than 2 cm, and 908 (57%) were found to be node-positive, as outlined in Table 1.

**Fig. 4:**
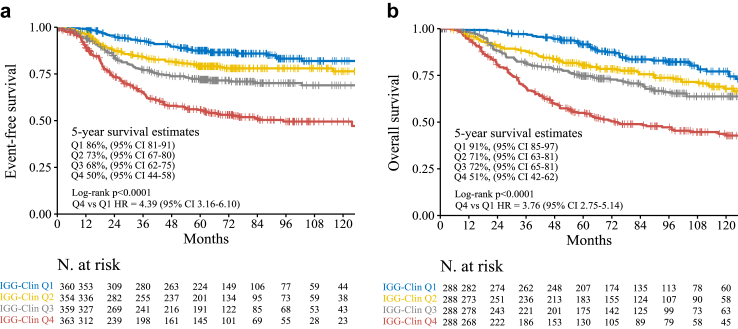
**Kaplan–Meier estimates in TNBC according to the expression of the IGG-Clin risk score (quartiles).** (a) EFS in the pooled dataset; (b) OS in the pooled dataset.

Analysing the IGG-Clin quartiles revealed significant prognostic implications for both EFS and OS. Compared to patients in the highest quartile, those in the lowest quartile experienced a notable 77% relative reduction in the risk of relapse or death (HR = 0.23, 95% CI = 0.16–0.32, p < 0.001), leading to a substantial 31% absolute improvement in EFS at the 5-year mark (Fig. 4A). Similarly, the analysis for OS showed promising outcomes, with patients in the lowest IGG-Clin quartile exhibiting a 52% reduction in the risk of death (HR = 0.52, 95% CI = 0.40–0.67, p < 0.001), translating to a remarkable 37% absolute increase in 5-year OS (Fig. 4B).

### Association of IGG with pCR

To assess the connection between IGG and pCR after neoadjuvant multi-agent chemotherapy, we examined the CALGB-40603 and BrighTNess datasets (see Table S4). The pCR rates were similar in both datasets: 48% (186/389) in CALGB-40603 and 49% (236/482) in BrighTNess.

**Fig. 5:**
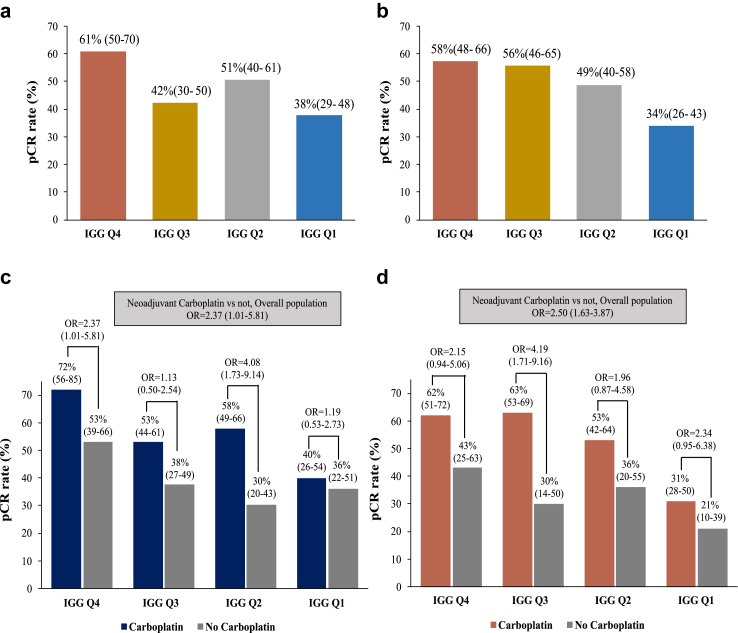
**Association of the IGG signature with pCR in CALGB-40603 and BrighTNess datasets.** (a, b) pCR rates according to the expression of the IGG signature according to quartiles in CALGB-40603 (a) and BrighTNess (b); (c, d) effect of carboplatin on pCR rates according to the expression of the IGG signature in (c) CALGB-40603 and (d) BrighTNess.

Our analysis revealed a significant association of IGG as a continuous variable with pCR in both the CALGB-40603 cohort (OR 1.25, [95% CIs 1.10–1.50], p = 0.006) and the BrighTNess cohort (OR 1.57 [1.25–1.98], p < 0.001). This association remained significant across different subgroups, including tumor size, nodal status, and carboplatin administration (Table 2). Furthermore, patients in the highest quartile of IGG expression exhibited significantly higher pCR rates compared to those in the lowest quartile in both CALGB-40603 (61% vs. 38%, OR = 2.56, 95% CIs = 1.45–4.60, p = 0.001) and BrighTNess (58% vs. 34%, OR = 2.64, 95% CIs = 1.57–4.48, p < 0.001]) cohorts (Fig. 5A and B and Table S5).

**Table 2:** Multivariable logistic regression analyses for pCR in CALGB-40603 and BrighTNess datasets.

Dataset	CALGB-40603		BrighTNess	
	HR (95% CI)	p value	HR (95% CI)	p value
IGG/immune	1.21 (1.10–1.53)	0.023	1.68 (1.32–2.15)	<0.0001
Tumour stage (T1 vs T2-4)	0.82 (0.42–1.50)	0.490	NA	NA
Nodal stage (N0 vs N+)	1.23 (0.80–1.91)	0.401	0.50 (0.32–0.74)	0.0003
Carboplatin (yes vs no)	1.90 (1.20–2.98)	0.003	2.70 (1.71–4.33)	<0.0001
Interaction IGG/immune∗carboplatin	–	0.800	–	0.20

NA, not available; HR, hazard ratio.

pCR improvement with carboplatin was noted across all quartiles of IGG (Fig. 5C and D), however, no significant interaction was observed between IGG as a continuous variable and carboplatin administration, both in CALGB-40603 (p-value for interaction = 0.800) and in BrighTNess (p-value for interaction = 0.200).

### Association of IGG with survival beyond pCR status and residual cancer burden

We investigated the relationship between IGG expression and survival outcomes in patients who underwent neoadjuvant multi-agent chemotherapy and had known pCR status. This evaluation was conducted using the CALGB-40603 dataset.^24^ The main results from CALGB-40603 trial (n = 443) did not show any significant association of tumor size and carboplatin with EFS, while pCR and nodal status were independent predictor of survival.^24^ These results were confirmed in our genomic dataset (n = 389). In the univariate analysis, nodal status (HR = 1.96, 95% CI = 1.29–2.97, p = 0.002), pCR (HR = 0.30, 95% CI: 0.20–0.46, p < 0.0001), and residual cancer burden (HR 1.37, 95% CI: 1.25–1.49, p < 0.0001) were significantly associated with EFS, while tumor size (HR = 0.81, 95% CI: 0.46–1.43, p = 0.469) and the use of carboplatin (HR = 0.78, 95% CI: 0.32–3.11, p = 0.498) were not. The IGG signature was also significantly associated with EFS (HR = 0.86, 95% CI = 0.75–0.99, p = 0.040).

Upon incorporating IGG, nodal status, and pCR status into a multi-variable model, all three variables continued to exhibit significant associations with EFS, including IGG (HR = 0.84, 95% CI: 0.72–0.98, p = 0.026). Likewise, IGG remained an independent predictor of improved EFS in a multi-variable model including nodal status and residual cancer burden (HR 0.83, 95% CI = 0.71–0.97, p = 0.017).

### Association of IGG-Clin with survival beyond pCR status and residual cancer burden

**Fig. 6:**
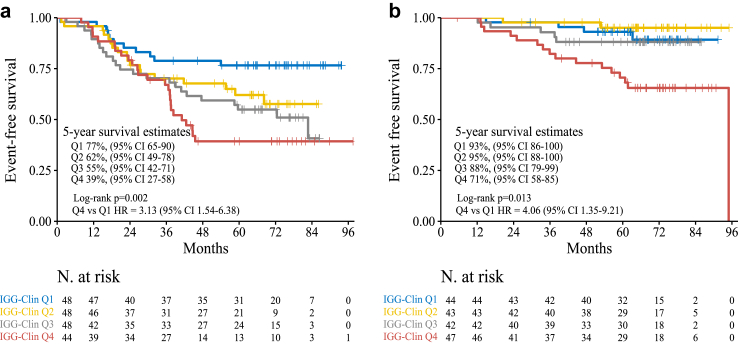
**Kaplan–Meier estimates in TNBC according to the IGG-Clin score and the type of pathological response in the CALGB-40603 trial.** EFS according to IGG-Clin score (quartiles) in patients with (a) residual disease or (b) pCR following neoadjuvant chemotherapy.

In the CALGB-40603 dataset, IGG-Clin, as a continuous variable, exhibited a significant association with EFS (HR = 3.22, 95% CI = 1.78–5.84, p < 0.001). When conducting bivariate analyses, considering pCR status and residual cancer burden (RCB), IGG-Clin remained significantly associated with survival (HR adjusted for pCR = 2.00, 95% CI = 1.47–2.66, p < 0.001; HR adjusted for RCB = 1.96, 95% CI = 1.40–2.74, p < 0.001). Within patients with residual disease and pCR, the IGG-Clin score effectively stratified individuals into different risk groups (Fig. 6A and B). In patients with a pCR, the IGG-Clin higher risk group (i.e., top-quartile) displayed a 5-year EFS estimate of 71% (95% CI = 58–85), while the lower risk group (bottom-quartile) displayed a 5-year EFS of 93% (95% CI = 86–100) (Fig. 6B).

### Relationship between IGG expression and immune cell subpopulations

**Fig. 7:**
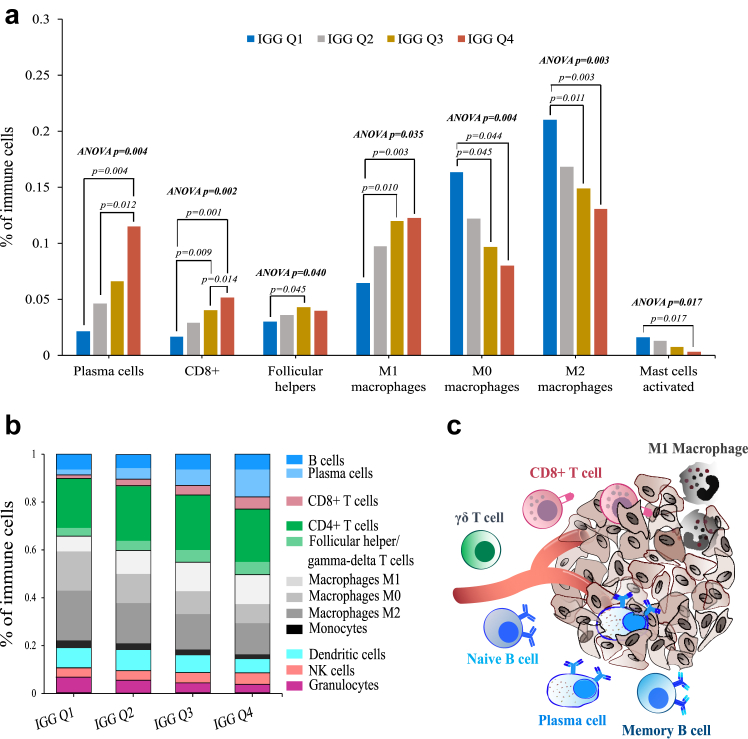
**Immune infiltrate composition in TNBC based on IGG expression in the SCAN-B, BrighTNess and CALGB-40603 datasets**. (a) Significant changes in immune cell types as determined by CIBERSORTx across IGG quartiles. Statistical differences were evaluated using a 2-way ANOVA considering both IGG quartiles and datasets, followed by Tukey's honest significant difference analysis; (b) Summary of the distribution of immune cell types as determined by CIBERSORTx across IGG quartiles. Percentages were calculated by averaging the expression of each cell type in each IGG quartile, with weighting based on the number of patients in each dataset; (c) Graphical representation of immune cells types associated with IGG in TNBC.

To explore the immunological features reflected by the IGG signature, each dataset was analysed through CIBERSORTx and Ecotyper deconvolution algorithms, in order to estimate the proportion or abundance of different immune cell types (CIBERSORTx) and immune activation states (EcoTyper) in the tumor microenvironment.^43^^,^^44^ The results showed that tumors in the top IGG quartile were significantly enriched for plasma cells (p = 0.004), CD8+ lymphocytes (p = 0.002), follicular helper T cells (0.040), and M1 macrophages (0.035), and significantly depleted of M0 and M2 macrophages (0.004 and 0.003, respectively) (Fig. 7, Figure S5, and Table S6). Accordingly, high IGG expression was significantly associated with immune ecotypes CE9 and CE10, which correspond to tumors with the highest levels of immunoreactivity of the tumor microenvironment and potential benefit from immunotherapy (2-way ANOVA p = 0.006 and p = 0.002, respectively) (Figure S6 and Table S7).

### Further relevance of the IGG signature

To further assess the relevance of the IGG signature in TNBC within the SCAN-B, CALGB-40603, and BrighTNess trials, we expanded our analysis and evaluated a panel of 185 breast cancer-related genes. This panel included the 14 IGG genes, the 50 genes of the PAM50 subtypes, and additional 121 genes linked to proliferation, luminal differentiation, HER2 signalling, and different immune processes (Table S8). From these 185 genes, only 10 showed consistent association with EFS in SCAN-B and CALGB-40603, and pCR in CALGB-40603 and BrighTNess (Figure S7). Notably, all 10 genes (thereafter called core immune genes or CIGs) are involved in immune response, with 5 (i.e., *CD79A, LAX1, PIM2, POU2AF1*, and *TNFRSF17*) being part of the IGG signature and tracking B-cell immunity. The observed overlap between the IGG signature and the identified CIGs is statistically significant and not expected by chance (p < 0.0001, exact hypergeometric probability test). As a proof of concept, we aimed to illustrate the improved prognostication achieved by amalgamating relevant immune genes into a unified signature, in contrast to the assessment of individual genes. We investigated whether a minimal signature composed of two CIGs could surpass the predictive and prognostic accuracy of individual CIGs. These immune signatures were constructed by applying addition, subtraction, multiplication, and division to the gene expression values. The prognostic and predictive information conveyed by each signature and gene was quantified using the chi-square (χ2) statistic obtained from a likelihood ratio test. Signatures generated by addition, mirroring the method used for the calculation of the IGG signature, exhibited superior prognostic and predictive information compared to single genes (Figure S8) and signatures derived from subtraction, multiplication, and division (Table S9).

## Discussion

In this study, we aimed to construct a prognostic score for TNBC utilising a combination of clinical and immune-genomic variables. We have successfully established that the IGG signature serves as an independent predictor of pCR and survival outcomes in early-stage TNBC. Building upon this discovery, we developed the IGG-Clin risk-score, which is a comprehensive assessment incorporating both the expression of the IGG signature and tumor stage. Notably, our study revealed that the IGG-Clin risk-score effectively stratifies patients into distinct risk groups with substantial variations in their likelihood of relapse.

After decades in which standard anthracycline/taxane-based chemotherapy has been the only option for TNBC, new approaches to either de-escalate or escalate treatment have now become available. These involve the exclusion of anthracyclines from platinum-taxane neoadjuvant regimens,^52^, ^53^, ^54^, ^55^, ^56^ the addition of immune checkpoint inhibitors (ICIs) and platinum agents to standard neoadjuvant chemotherapy,^57^ adjuvant capecitabine to treat residual disease in the post-neoadjuvant setting,^58^ and adjuvant olaparib in high-risk patients with germline *BRCA1/2* mutations.^59^ Currently, there is a lack of standardized prognostic tools that enable personalized treatment selection, leading to the reliance on tumor stage assessment in the pre-treatment context and evaluation of tumor response in the post-neoadjuvant setting for patient selection. However, it is crucial to recognize that tumor biology plays a substantial role in determining patients' outcomes. In stage I disease, historical series report recurrence rates between 5 and 30%, underlining prognostic heterogeneity in patients with low clinical risk, and the need of novel biomarkers to identify those who may benefit from escalated approaches.^60^^,^^61^

According to our study, IGG expression discriminated between tumors with excellent (5-years relapse risk <10%) and dismal prognosis (5-years relapse risk ∼20%) within stage I TNBC, and between those at high (>30%), intermediate (15%) and lower (∼10%) risk of relapse within the T1-2 N0 group. Therefore, this signature could represent a valuable biomarker to stratify patients with low tumor burden. In stage II-III disease, recurrence rates are estimated to be ≥ 30–40%, a sufficient risk to recommend treatment escalation with neoadjuvant carboplatin and ICIs.^4^^,^^50^ However, evidence indicates that some patients could be cured with less intense treatments. In the control arms of neoadjuvant immunotherapy trials, 77–81% of participants remained free of recurrence at 3 years with chemotherapy alone.^57^^,^^62^, ^63^, ^64^ Similarly, neoadjuvant trials assessing the addition of platinum agents show that up to 70% of patients with stage II-III TNBC remain free of recurrence with standard anthracycline-based chemotherapy.^49^^,^^51^ Finally, phase II trials show that 12–18 cycles of taxane-carboplatin are sufficient to achieve pCR in 49–55% of stage I-III TNBCs, suggesting that de-escalation might be an option for some patients.^52^, ^53^, ^54^, ^55^, ^56^

In this scenario, combining the prognostic information conveyed by IGG expression and tumor burden could improve patient selection. The IGG-Clin algorithm re-classified patients in 4 groups with large differences in their risk of relapse and death. With a 50% probability of recurrence at 5 years, the highest risk group would be the most likely to benefit from combined escalated approaches, such as the addition of both carboplatin and ICIs to NACT. Patients in lower risk groups might benefit instead from intermediate approaches, whose toxicities do not outbalance potential benefits. A biomarker analysis of the NeoPACT trial showed that patients in the IGG-Clin low-risk group have excellent outcomes with the anthracycline-free regimen of carboplatin/docetaxel plus pembrolizumab (3-year EFS of 93%), while for IGG-Clin high-risk patients the prognosis is less favorable with such de-escalated approach (3-EFS of 80%).^65^ Of note, the same analysis also showed that tumors with high IGG expression (above median) achieve pCR rates of 70% with the NeoPACT regimen, while in low IGG tumors, the rate dropped to 43%.^65^

In the post-neoadjuvant setting, patients with residual disease are currently endorsed for adjuvant systemic treatments, while no additional therapy is recommended for patients with pCR.^58^^,^^59^^,^^66^ In this framework, novel biomarkers like RCB are being proposed to improve upon such dichotomous distinction.^67^^,^^68^ In CALGB-40603, IGG expression and IGG-Clin risk score were associated with survival outcomes independently of pCR status and RCB, suggesting that IGG-based biomarkers might add prognostic information beyond the evaluation of pathological response. IGG-Clin identified patients with pCR at substantial risk of relapse (∼30% at 5 years), who might benefit from adjuvant treatments despite the achievement of a complete response upon NACT.

From a biological standpoint, our findings highlight the significant role of B-cell immunity in the prognosis of TNBC. While B-lymphocytes represent a minor component of TILs, their presence could indicate the activation of an antigen-driven immune response against tumor cells.^69^ In a study conducted by Shepherd and colleagues in the CALGB-40603 trial,^24^ oligoclonal expansion of IgG-biased B-lymphocytes within the tumor infiltrate was highly prognostic. Similar results have been observed in another retrospective series.^22^

By employing immune deconvolution analysis with CIBERSORTx and EcoTyper, we made a significant observation regarding TNBCs with heightened IGG expression. We found an enrichment of various cell types involved in the initiation, regulation, and maintenance of adaptive immune responses. Among these cell types were plasma cells, B memory lymphocytes, gamma-delta T lymphocytes, CD8+ lymphocytes, and M1 macrophages. Remarkably, similar results have been reported recently in the NeoPACT trial,^65^ reinforcing the validity of our findings.

Our study has a few noteworthy limitations. Overall, datasets exhibited significant heterogeneity in terms of clinical data and patient characteristics. A limitation in terms of internal validity is the use of different surrogate survival endpoints across datasets. While this variability could have potentially impacted the association of IGG and IGG-Clin with the risk of recurrence, it is worth noting that the OS analysis consistently demonstrated a strong association of these biomarkers with patients’ survival, reaffirming their prognostic significance. Methodological limitations such as the small number of studies included in the random-effects models, unmeasured confounding and a potential selection bias in HRs estimation should also be acknowledged.^70^ Additionally, genomic profiling was conducted using different platforms across the datasets, making it challenging to create a unified dataset with continuous gene expression information. Consequently, several factors could have contributed to the lack of a significant prognostic effect of the IGG signature in the TCGA and GSE58812 datasets, including the limited sample size, shorter follow-up periods, and variations in gene expression analysis methodologies. From a clinical standpoint, the main limitation is the unavailability of TILs data in patients with survival or pCR information, which precluded to assess whether the addition of IGG would enhance the prognostication of TNBC in conjunction with TILs. Patients with node-negative TNBCs high in TILs (≥75%) have shown excellent survival outcomes without (neo)adjuvant systemic therapy, making TILs a valuable biomarker for potential treatment de-escalation.^12^ Whether immune genomic signatures could outperform TILs in selecting patients for treatment de-escalation and escalation is currently a matter of research. Recently, a correlative analysis of the CALGB-40601 and the PAMELA trial showed that in HER2-positive early breast cancer the IGG signature outperformed TILs for both pCR and prognosis.^40^ Similar analyses are warranted in trials including patients with TNBC to compare the prognostic ability of these different means of measuring immune activation. Finally, in the CALGB-40603 and BrighTNess trials, we did not observe any association of IGG with carboplatin response. However, it is important to note that these trials involved the administration of additional investigational treatments (bevacizumab and veliparib, respectively), which could have acted as confounding factors impacting the results.

To conclude, this study presents groundbreaking evidence indicating the potential for enhanced prognostication in TNBC through the utilisation of multi-feature classifiers that combine immune biomarkers and clinical data. Furthermore, our findings contribute to the expanding body of research emphasizing the crucial involvement of B-cell immunity in the context of TNBC.

## Contributors

Aleix Prat, Benedetta Conte, and Fara Brasó-Maristany contributed to the conceptualization of the study. Benedetta Conte wrote the original draft of the manuscript, with supervision provided by Aleix Prat and Fara Brasó-Maristany. Data curation was handled by Aleix Prat, Benedetta Conte, Fara Brasó-Maristany, Laia Paré, Tomás Pascual, Guillermo Villacampa, Adela Rodríguez Hernández, Francesco Schettini, Maria J. Vidal Losada, Elia Seguí, Laura Angelats, Isabel Garcia-Fructuoso, Raquel Gomez-Bravo, Natàlia Lorman-Carbó, Mercedes Marín-Aguilera, Olga Martínez-Sáez, Barbara Adamo, Esther Sanfeliu, Beatrice Fratini, Claudette Falato, Núria Chic, Ana Vivancos, Patricia Villagrasa González, Johan Staaf, Joel S. Parker, and Charles M. Perou. The methodology was designed by Aleix Prat, Benedetta Conte, and Fara Brasó-Maristany, with the formal analysis being performed by them. Guillermo Villacampa reviewed the formal analysis. All authors contributed to the review and editing of the manuscript. Aleix Prat, Fara Brasó-Maristany, and Laia Paré verified the underlying data (validation). All authors have read and agreed to the published version of the manuscript.

## Data sharing statement

All genomic and clinical data used in this paper can be retrieved from public repositories. The Cancer Genome Atlas (TCGA) and METABRIC data were retrieved from the cBio Cancer Genomics Portal (http://cbioportal.org). Data from the last updated version of the SCAN-B dataset was retrieved from Mendeley Data (https://data.mendeley.com/datasets/yzxtxn4nmd). Data from GSE21653, GSE58812 and BrighTNess (accession number GSE164458) were retrieved from the NCBI Gene Expression Omnibus (GEO) repository (https://www.ncbi.nlm.nih.gov). Data from CALGB-40603 were obtained from the dbGap repository.

## Declaration of interests

B. Conte reports speaker fees from Veracyte and payment for educational events from Medsite and Novartis. A. Prat reports consulting fees from Roche, Pfizer, Novartis, Amgen, BMS, Puma, Oncolytics Biotech, MSD, Guardant Health, Peptomyc and Lilly, lecture fees from Roche, Pfizer, Novartis, Amgen, BMS, Nanostring Technologies and Daiichi Sankyo; patents filed PCT/EP2016/080056, PCT/EP2022/086493, PCT/EP2023/060810, EP23382703 and EP23383369; stockholder and consultant of Reveal Genomics, SL; and institutional financial interests from Boehringer, Novartis, Roche, Nanostring, Sysmex Europa GmbH, Medica Scientia Innovation Research, SL, Celgene, Astellas and Pfizer. F. Schettini has declared consulting fees from Pfizer, honoraria for lectures from Novartis, Gilead and Daiichy Sankyo, and travel expenses from Novartis and Daiichy Sankyo. O. Martínez-Sáez has declared institutional grants from Roche; personal consulting fees from Roche and Reveal Genomics; honoraria for presentations by Daiichi Sankyo, Pierre Fabre, and Reveal Genomics; and travel expenses by Gilead, Pierre Fabre, Novartis, and MSD. A. Vivancos has declared institutional grants from Bristol Meyers Squibb, Incyte, and Roche; personal consulting fees from Bayer, Bristol Meyers Squibb (BMS); Guardant, Incyte, and Roche; and personal stock options from Reveal Genomics. F. Brasó-Maristany has patents filed: PCT/EP2022/086493, PCT/EP2023/060810, EP23382703 and EP23383369. J. Parker as declared individual and institutional royalties from Veracyte–Prosigna, consulting fees from Bristol Meyers Squibb, Reveal Genomics, and GeneCentric, and holds a patent for breast cancer subtyping. Additionally, he has an equity interest in Reveal Genomics. M. Vidal has declared honoraria for presentations from Novartis, Roche, Pfizer, and Daichii, and travel expenses from Roche and Pfizer. Additionally, she has participated on a Data Safety Monitoring Board or Advisory Board for Novartis and Roche. C. Perou has declared consulting fees and personal stock options from Reveal Genomics. T. Pascual has declared consulting fees from Novartis; honoraria from Novartis, Astra-zeneca, Veracyte, and Argenetics. I. Garcia-Fructuoso has declared honoraria for presentations from Novartis, Daiichi Sankyo, Esteve, GSK; and travel expenses from Novartis, Gilead, Daiichi Sankyo, Lilly, and BMS. L. Paré has declared contract from Reveal Genomics, a HER2DX patent filed (PCT/EP2022/086493), and the TNBCDX patent filed (EP23382703.9). M. Marín-Aguilera has declared contract from Reveal Genomics. P. Villagrasa has declared contract and personal stock options from Reveal Genomics, the HER2DX patent filed (PCT/EP2022/086493), and the DNADX patent filed (EP22382387.3). G. Villacampa has received a speaker's fee from Merck Sharp & Dohme, Pfizer, GlaxoSmithKline and Pierre Fabrer, and received consultant fees from Reveal Genomics. C. Falato is currently employed in AstraZeneca.

The remaining authors declare no competing interests.
