# Supplementary material for: A 14-gene B-cell immune signature in early-stage triple-negative breast cancer (TNBC): a pooled analysis of seven studies
Source: eBioMedicine. 2024 Mar 5;102:105043. doi: 10.1016/j.ebiom.2024.105043 (PMC10924177; doi:10.1016/j.ebiom.2024.105043)
Supplement: Supplementary Figures and Tables [file mmc1.docx]

Contents

[Supplementary methods 3](#_Toc151652923)

[Figure S1 8](#_Toc151652924)

[Figure S2. 9](#_Toc151652925)

[Figure S3. 10](#_Toc151652926)

[Figure S4. 11](#_Toc151652927)

[Figure S5. 12](#_Toc151652928)

[Figure S6. 13](#_Toc151652929)

[Table S3 16](#_Toc151652930)

[Table S4. 16](#_Toc151652931)

[Table S7 22](#_Toc151652932)

# Supplementary methods

**Accesion and analysis of gene expression data**

Gene expression data were directly downloaded from the following links:

METABRIC: <https://www.cbioportal.org/results/download?cancer_study_list=brca_metabric&Z_SCORE_THRESHOLD=2.0&RPPA_SCORE_THRESHOLD=2.0&profileFilter=mrna_median_all_sample_Zscores&case_set_id=brca_metabric_all&gene_list=CXCL8%250ANTN3%250AHLA-C%250AIL2RG%250ACD27%250APOU2AF1%250ALAX1%250ACD79A%250APIM2%250ATNFRSF17%250AIGKC%250AJCHAIN%250AIGL%250AIGLV3-25&geneset_list=%20&tab_index=tab_visualize&Action=Submit>

TCGA:

<https://www.cbioportal.org/results/download?cancer_study_list=brca_tcga_pan_can_atlas_2018&Z_SCORE_THRESHOLD=2.0&RPPA_SCORE_THRESHOLD=2.0&profileFilter=rna_seq_v2_mrna_median_Zscores&case_set_id=brca_tcga_pan_can_atlas_2018_rna_seq_v2_mrna&gene_list=CXCL8%250ANTN3%250AHLA-C%250AIL2RG%250ACD27%250APOU2AF1%250ALAX1%250ACD79A%250APIM2%250ATNFRSF17%250AIGKC%250AJCHAIN%250AIGL%250AIGLV3-25&geneset_list=%20&tab_index=tab_visualize&Action=Submit>

BrighTNess: <https://www.ncbi.nlm.nih.gov/geo/query/acc.cgi?acc=GSE164458>

CALGB-40603: <https://www.ncbi.nlm.nih.gov/projects/gap/cgi-bin/study.cgi?study_id=phs001863.v1.p1>

GSE21653: <https://ftp.ncbi.nlm.nih.gov/geo/series/GSE21nnn/GSE21653/matrix/>

GSE58812: <https://ftp.ncbi.nlm.nih.gov/geo/series/GSE58nnn/GSE58812/matrix/>

Z-score transformed values were used for METABRIC and TCGA, while normalized data were downloaded for SCANB, BrighTNess, CALGB-40603, GSE58812, and GSE21653. Normalized data were log2-transformed before signature calculation.

**Testing for normality and homogeneity of variance for 2-way ANOVA and Tukey’s test**

Differences in immune cell-type composition between IGG groups (i.e., quartiles), were compared using a 2-way ANOVA considering both IGG quartiles and datasets, followed by Tukey’s honest significant difference analysis. Anderson-Darling test and Levene’s test were used to assess for normality and homogeneity of variance, respectively (table1). Figure 1 shows the Q-Q plot of residuals for each cell population derived from the CIBERSORTx analysis. Except for Dendritic cells resting, NK activated and resting cells, T gamma-delta cells, Eosinophils, and B memory cells, the data for other cell populations met the normality assumption, and all cells show homogeneity of variance. Moreover, Figure 1's Q-Q plots illustrate that the residual distributions do not substantially deviate from normality, suggesting that our analyses are not unduly influenced by non-normality

**Table 1**. P values from the Anderson-Darling test and Levene’s test for homogeneity of variance for each cell population. Both tests were performed on the output obtained from the CIBERSORTx deconvolution analysis.

| Cell type | Anderson-Darling test  (p value) | Levene's Test for Homogeneity  of Variance (p value) |
| --- | --- | --- |
| Dendritic.cells.resting | 0.0001 | 0.99 |
| NK.cells.activated | 0.0003 | 0.97 |
| T.cells.gamma.delta | 0.0008 | 0.59 |
| Eosinophils | 0.0049 | 0.47 |
| B.cells.memory | 0.010 | 0.97 |
| NK.cells.resting | 0.028 | 0.96 |
| Monocytes | 0.071 | 0.96 |
| Neutrophils | 0.074 | 0.92 |
| T.cells.CD4.naive | 0.074 | 0.99 |
| Dendritic.cells.activated | 0.13 | 0.70 |
| Mast.cells.activated | 0.14 | 0.66 |
| Mast.cells.resting | 0.17 | 0.74 |
| Plasma.cells | 0.26 | 0.75 |
| Macrophages.M1 | 0.35 | 0.78 |
| T.cells.regulatory..Tregs. | 0.36 | 0.86 |
| Macrophages.M2 | 0.38 | 0.92 |
| T.cells.follicular.helper | 0.49 | 0.95 |
| T.cells.CD4.memory.resting | 0.60 | 0.99 |
| T.cells.CD4.memory.activated | 0.68 | 0.51 |
| Macrophages.M0 | 0.77 | 0.99 |
| B.cells.naive | 0.80 | 0.75 |
| T.cells.CD8 | 0.85 | 0.84 |

**Figure 1**. Q-Q plots of residuals from the Anderson-Darling test for normality for each cell type.


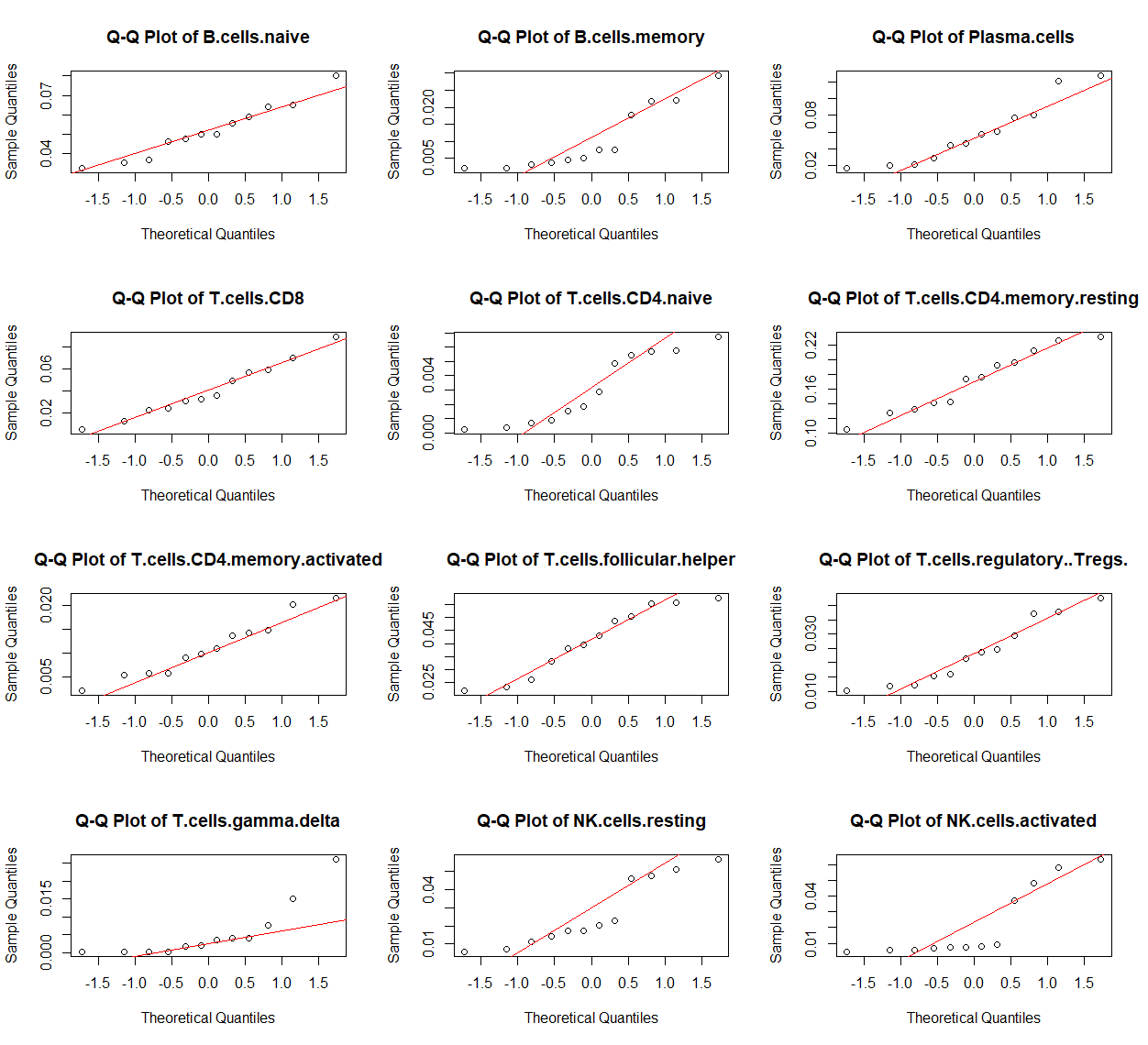


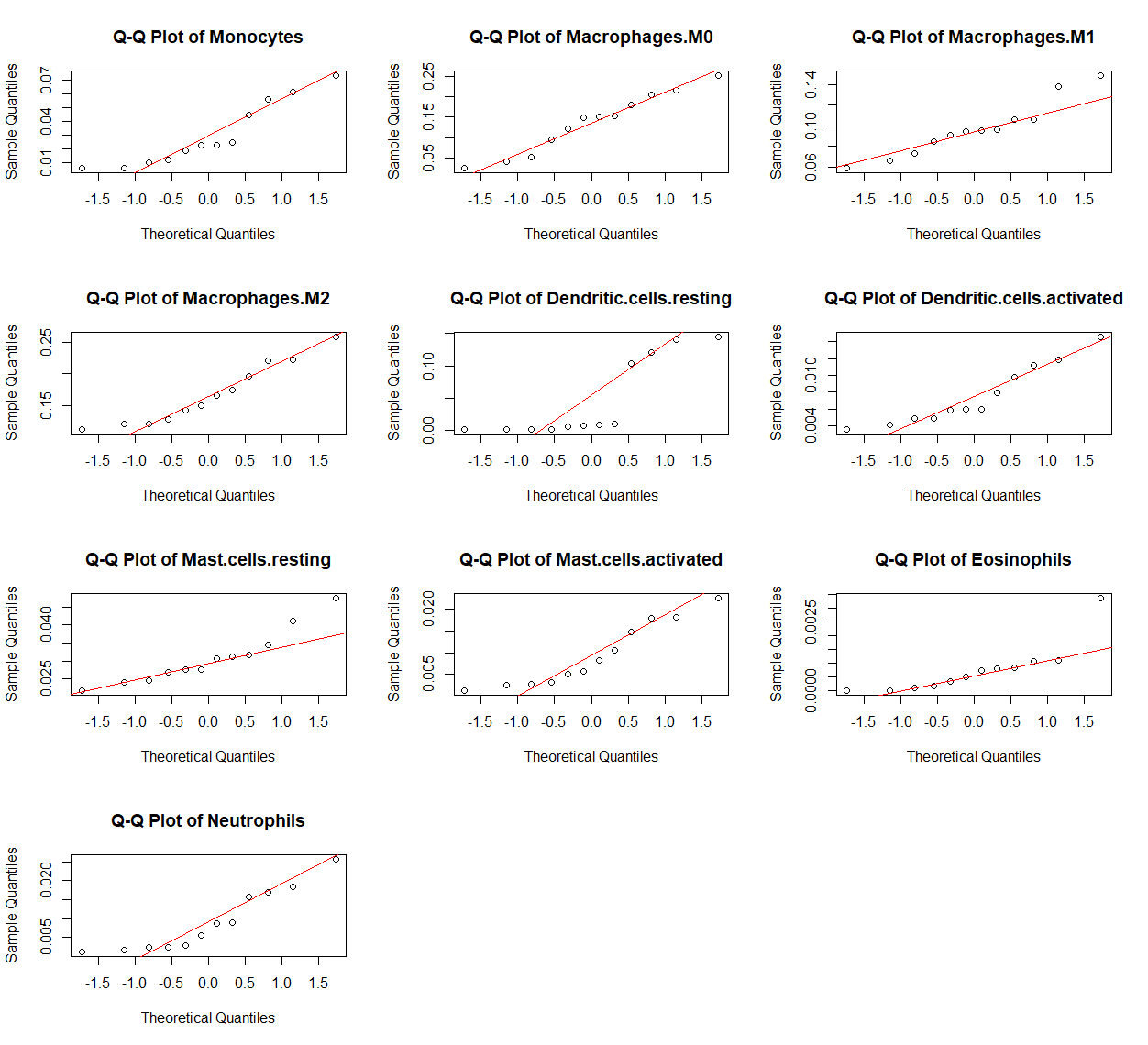


**Testing proportional hazard and linearity assumptions for Cox and logistic regression models**

Cox’s proportional hazard assumption for each variable was assessed and confirmed through Schoenfeld’s individual test in each dataset, as depicted in figure 2 and table 2. To assess linearity, these models were fitted with the continuous predictors either entered linearly or adjusted for potential non-linearity. For the latter, we used the “pspline” function in R to fit a penalized spline with four degrees of freedom (df=4) to allow for adequate flexibility while avoiding overfitting. Then, a likelihood ratio test between the linear and non-linear models was performed to check significant deviance from the null hypothesis for linearity. Results from likelihood ratio tests are depicted in table 3. The p-values from these tests, as shown in Table 3, demonstrate that for the majority of datasets, there is no evidence to reject the linearity assumption.

**Figure 2**. Schoenfeld’s residuals distribution for IGG for Cox univariate models for EFS and OS across datasets.


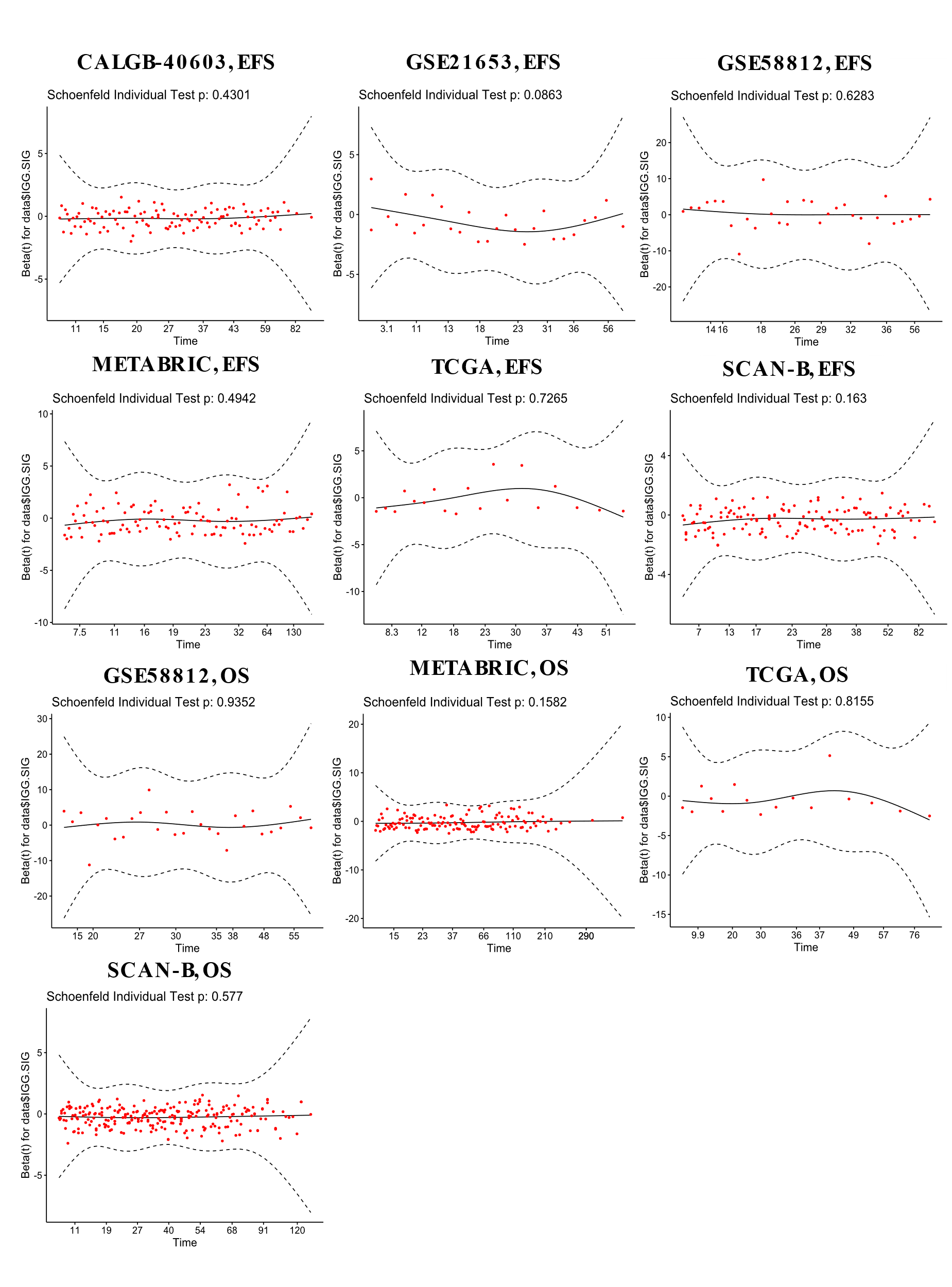


**Table 2**. Significance of Schoenfeld’s individual test for covariates included in Cox multivariate models for EFS and OS across datasets.

| **Dataset** | **Covariate** | **Cox Model** | |
| --- | --- | --- | --- |
|  |  | **EFS** | **OS** |
| CALGB-40603 | Tumor size (T1 vs T2-4) | 0.91 | NA |
|  | Nodal status (N0 vs N+) | 0.19 | NA |
|  | pCR (yes vs no) | 0.04 | NA |
|  | RCB class (0 vs 1 vs 2 vs 3) | 0.12 | NA |
| GSE21653 | Tumor size (T1 vs T2-4) | 0.61 | NA |
|  | Nodal status (N0 vs N+) | 0.85 | NA |
| GSE58812 | Tumor size (T1 vs T2-4) | NA | NA |
|  | Nodal status (N0 vs N+) | NA | NA |
| METABRIC | Tumor size (T1 vs T2-4) | 0.83 | 0.49 |
|  | Nodal status (N0 vs N+) | 0.72 | 0.007 |
| TCGA | Tumor size (T1 vs T2-4) | 0.30 | 0.63 |
|  | Nodal status (N0 vs N+) | 0.22 | 0.02 |
| SCAN-B | Tumor size (T1 vs T2-4) | <0.0001 | 0.002 |
|  | Nodal status (N0 vs N+) | 0.048 | 0.002 |

**Table 3**. P values from likelihood ratio tests between the linear and non-linear Cox and logistic univariate regression models for the association of IGG signature with EFS, OS and pCR. P values >0.05 indicate models including IGG signatures as continuous expression do not deviate significantly from the linearity assumption at an alpha level of 0.05.

| **Dataset** | **Cox univariate model** | | **Univariate Logisic Regression Model** |
| --- | --- | --- | --- |
|  | **EFS** | **OS** | **pCR** |
| CALGB-40603 | 0.085 | NA | 0.65 |
| BrighTNEss | NA | NA | 0.38 |
| GSE21653 | 0.11 | NA | NA |
| GSE58812 | 0.087 | 0.13 | NA |
| METABRIC | 0.47 | 0.17 | NA |
| TCGA | 0.26 | 0.65 | NA |
| SCAN-B | 0.31 | 0.39 | NA |

**IGG expression distribution across demographic and clinical-pathological factors**

For each dataset, we evaluated the distribution of the IGG signature (continuous expression), across demographic and clinical-pathological characteristics. As depicted in Figure 3a, high, intermediate, and low levels of IGG expression were found across all age categories and were not exclusive to specific clinical-pathological status. Figure 3b shows no major changes in IGG expression across ethnicity in two additional datasets (TCGA Firehose version and CPTAC dataset).


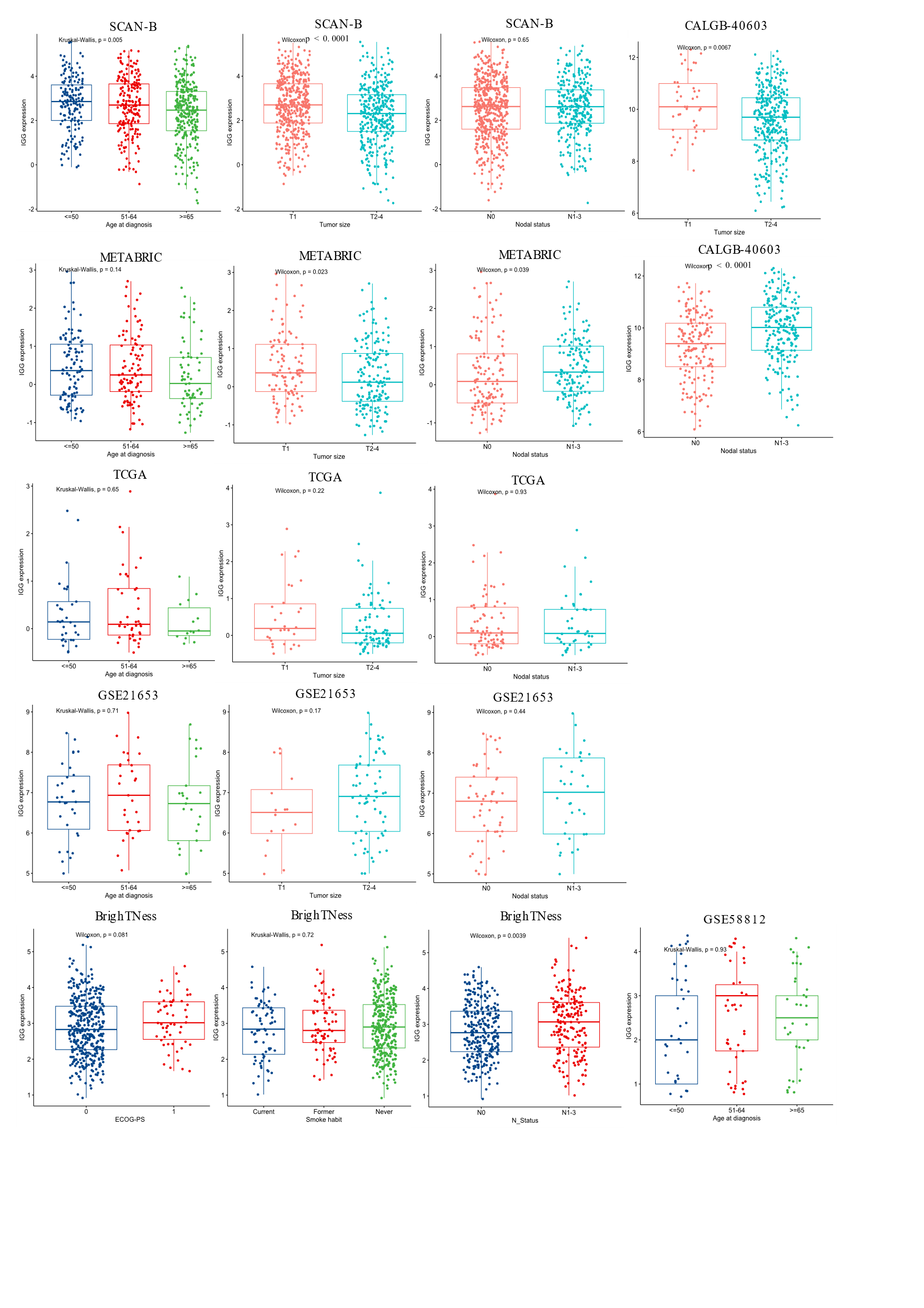
**Figure 3a.** IGG expression across demographic and clinical-pathological factors in each dataset

**Figure 3b.** IGG expression distribution across different ethnicities in the TCGA Firehose^1^ and CPTAC^2^ datasets.


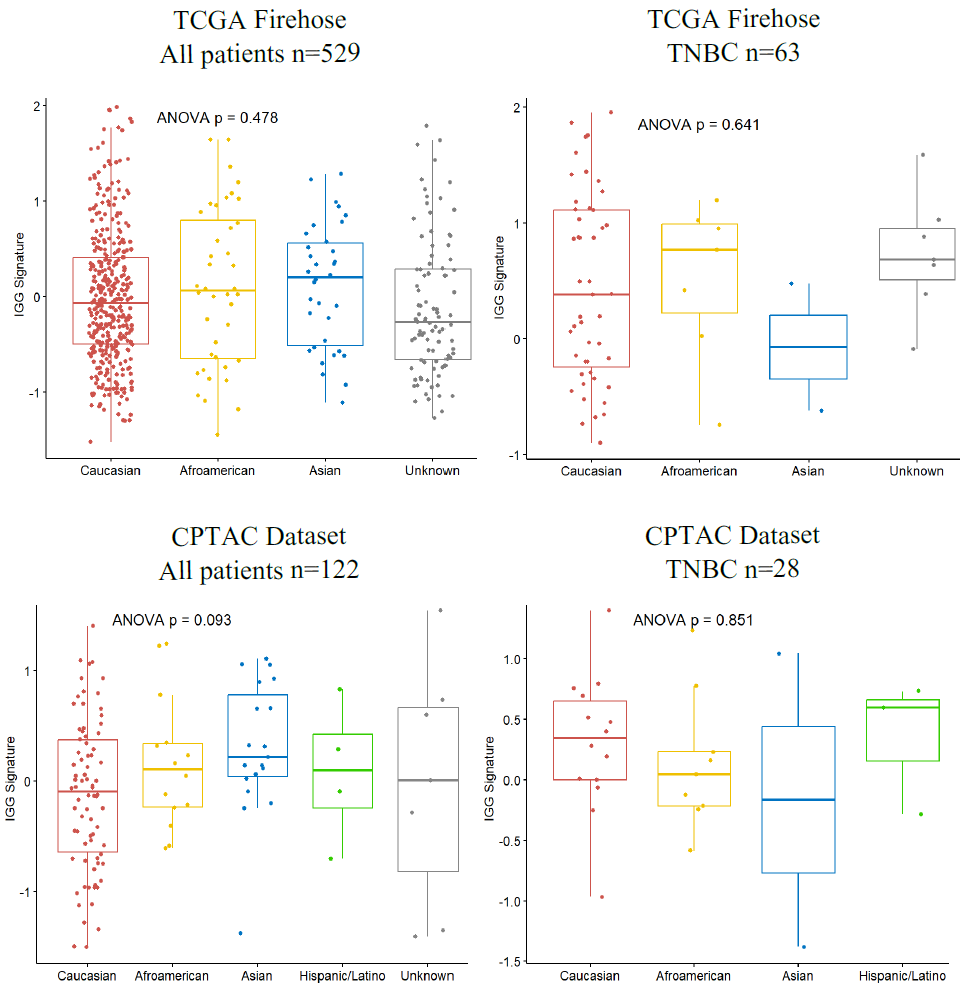


^1^ Source: <https://www.cbioportal.org/study/summary?id=brca_tcga>

^2^ Krug K, Jaehning EJ, Satpathy S, et al. Proteogenomic Landscape of Breast Cancer Tumorigenesis and Targeted Therapy. Cell 2020; 183(5):1436-1456.e31.

**Consistency of IGG expression across genomic platform and justification for IGG-based prognostic groups**

Gene expression was evaluated with different methods across dataset. In METABRIC and TCGA datasets gene expression was available in the form of z-score transformed data obtained from RNA-seq (TCGA) or Illumina Microarray (METABRIC) platforms. In others datasets, gene expression was available in the form of log2 transformed RNA-seq (SCAN-B, CALGB-40603, BrighTNess) or Affymetrix Microarray data (GSE21653, GSE58812). The table and scatter plots below show the correlation between datasets in terms of average expression of the 14 genes from the IGG signature. Overall, these results show moderate to high correlation between z-score transformed data, obtained either with RNA-seq (TCGA) or with Illumina Microarray (METABRIC), and log2-tranformed data obtained with RNA-seq (SCAN-B, CALGB-40603, BrighTNess). However, the correlation is low in the case of log2-tranformed Affymetrix Microarray data (GSE21653, GSE58812). Moreover, while top expressed genes (PIM2, ILRG2) and the least expressed ones (NTN3) are consistent across platforms, the expression of specific genes showed remarkable inconsistency across platforms (i.e. HLA-C). Due to such heterogeneity, the identification of a standardized uniform cutoff for IGG expression for prognostic prediction was not feasible. Instead, prognostic groups were determined by arbitrarily dividing patients into IGG expression quartiles. This quartile-based stratification enabled appropriate discrimination between the extremes of expression levels (i.e., lowest quartile of expression versus top quartile of expression). Additionally, it maintained a degree of homogeneity within the combined dataset post-merger.

**Table 4.** Spearman’s correlation coefficients between datasets in terms of average expression of the 14 genes from the IGG signature.

|  | **Z-score transformed** | | **Log2-tranformed** | | | | |  |
| --- | --- | --- | --- | --- | --- | --- | --- | --- |
|  | **gene expression** | | **gene expression** | | | | |  |
|  | **RNA-seq** | **Illumina Microarray** | **RNA-seq** | **RNA-seq** | **RNA-seq** | **Affymetrix Microarray** | **Affymetrix Microarray** |  |
|  |  |  |  |  |  |  |  |  |
|  | **TCGA** | **METABRIC** | **SCANB** | **BrighTNess** | **CALGB 40603** | **GSE21653** | **GSE58812** |  |
| **TCGA** | **1** |  |  |  |  |  |  |  |
| **METABRIC** | **0.66** | **1** |  |  |  |  |  |  |
|  | **(0.10-0.90)** |  |  |  |  |  |  |  |
| **SCANB** | **0.45** | **0.36** | **1** |  |  |  |  |  |
|  | **(0.35-0.91)** | **(0.29-0.98)** |  |  |  |  |  |  |
| **BrighTNess** | **0.1** | **0.2** | **0.84** | **1** |  |  |  |  |
|  | **(-0.3-0.36)** | **(-0.1-0.60)** | **(0.45-0.96)** |  |  |  |  |  |
| **CALGB 40603** | **0.52** | **0.52** | **0.9** | **0.98** | **1** |  |  |  |
|  | **(0.32-0.89)** | **(0.21-0.94)** | **(0.39-0.97)** | **(0.87-0.99)** |  |  |  |  |
| **GSE21653** | **0.16** | **0.05** | **0.89** | **0.87** | **0.9** | **1** |  |  |
|  | **(-0.66-0.36)** | **(-0.51-0.70)** | **(0.58-0.98)** | **(0.23-0.95)** | **(0.63-0.98)** |  |  |  |
| **GSE58812** | **0.07** | **-0.11** | **0.72** | **0.43** | **0.56** | **0.5** | **1** |  |
|  | **(-0.9-0.50)** | **(-0.8-0.70)** | **(0.16-0.86)** | **(0.03-0.70)** | **(0.15-0.88)** | **(0.15-0.81)** |  |  |

**Figure 4.** Scatter plots showing the correlation between the z-score transformed average expression of the 14 genes composing the IGG signature in METABRIC and TCGA datasets, and the log2-transformed average expression of the same genes in SCAN-B, GSE21653 and GSE58812.


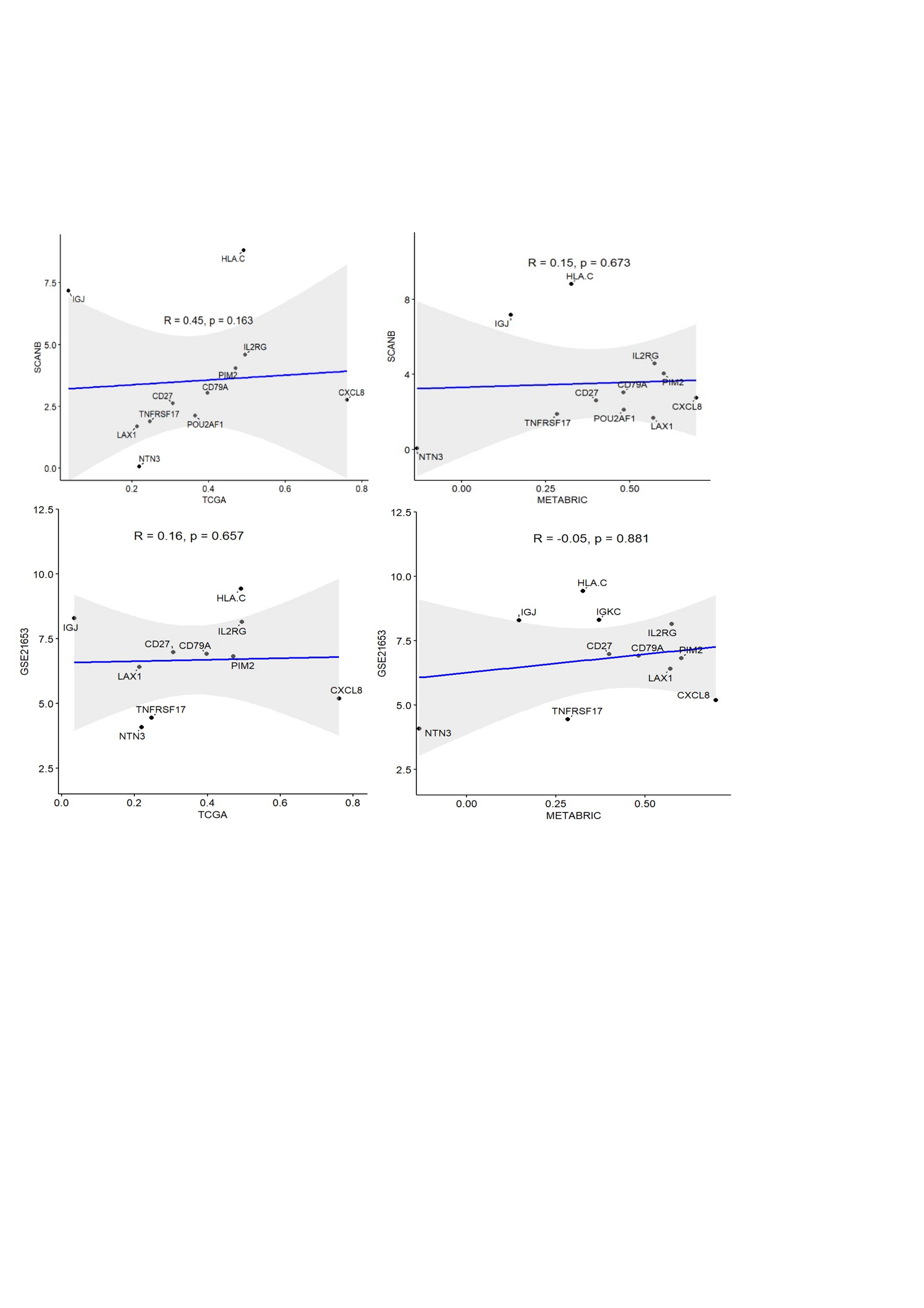


Figure S1. **Composition of IGG genes across datasets.** The figure illustrates the presence of genes in blue color within each dataset, while absent genes are indicated in grey.

**
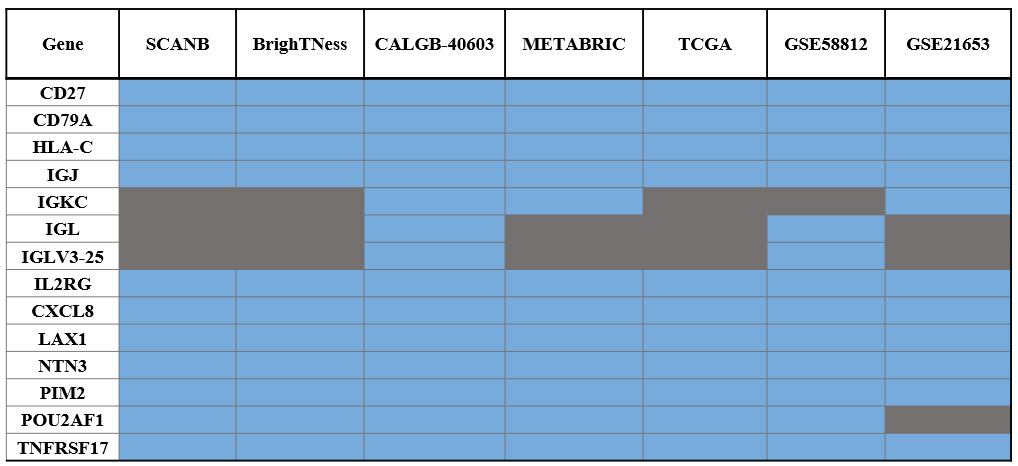
**

Figure S2. **Hazard ratios and pooled effect sizes concerning the association of IGG with survival outcomes**. **(a)** Unadjusted Hazard Ratios (HRs) for Event-Free Survival (EFS); **(b)** Unadjusted HRs for Overall Survival (OS); **(c)** Adjusted HRs for EFS; **(d)** Adjusted HRs for OS. Adjusted HRs were computed by incorporating age (continuous), tumor stage (T1 vs. T2-4), nodal status (N0 vs. N+), and (neo)adjuvant treatment (no systemic treatment vs. chemotherapy vs. unknown) into the Cox model. The square sizes are proportionate to the weights assigned to each dataset.


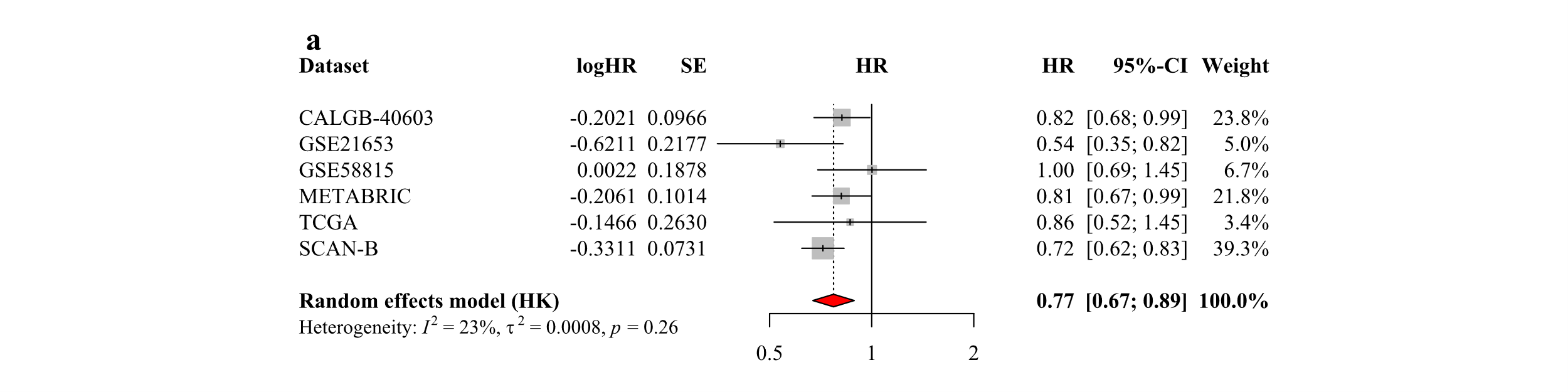


**
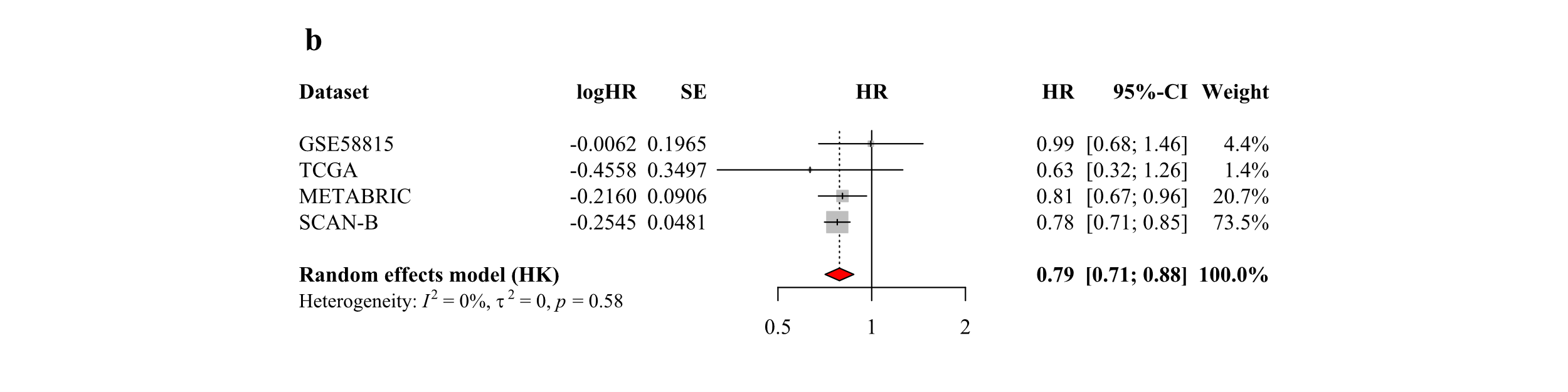
**


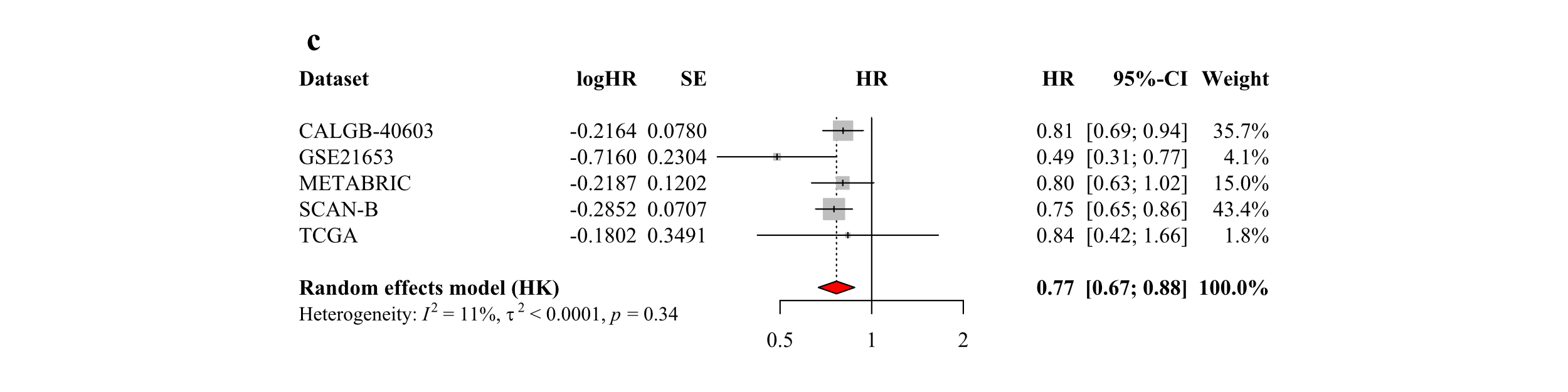


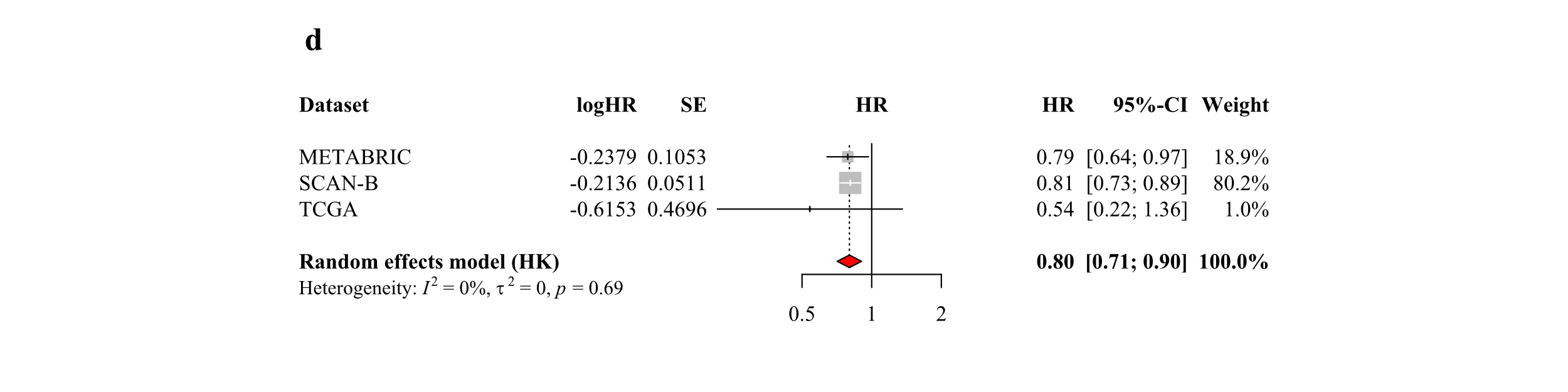


HR, hazard ratio; 95%-CI, 95% confidence intervals.


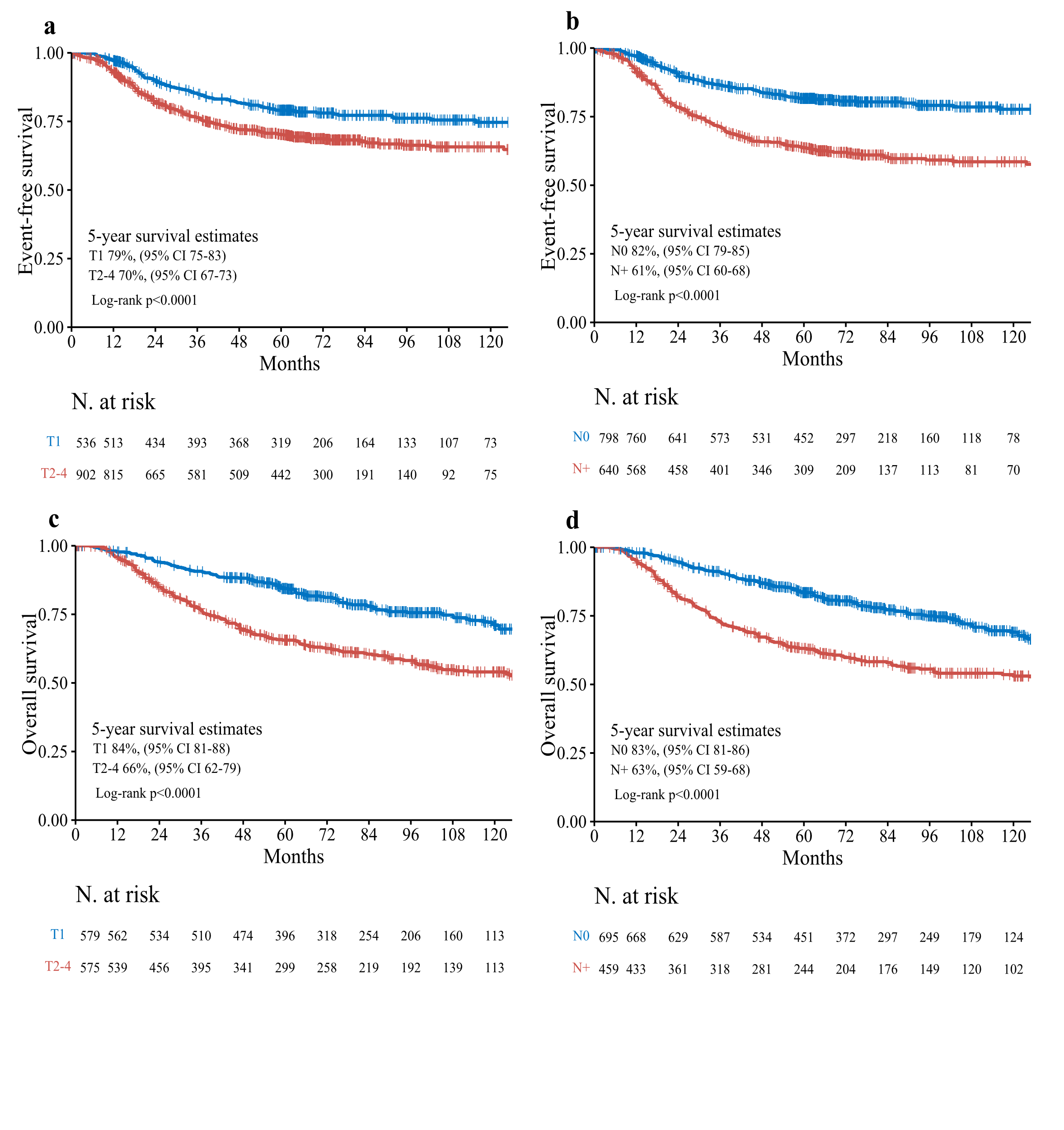
Figure S3. **Kaplan-Meier estimates according to tumor stage. (a)** EFS according to tumor size (T1 versus T2-4); **(b)** EFS according to nodal status (N0 versus N+); **(c)** OS EFS according to tumor size (T1 versus T2-4); **(d)** OS according to nodal status (N0 versus N+).


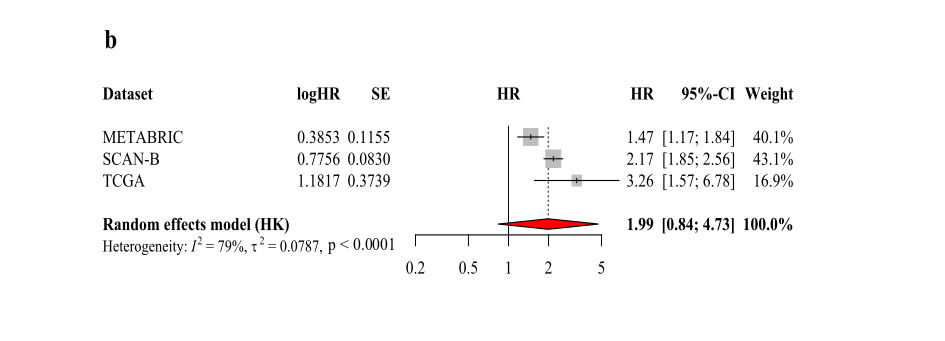

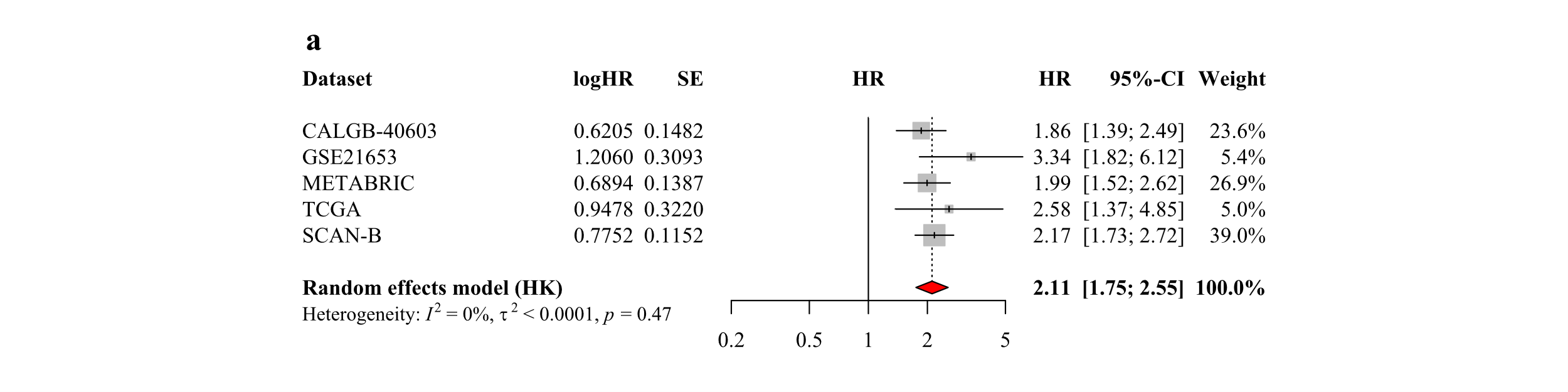
Figure S4. **Hazard ratios and pooled effect sizes to assess the relationship between IGG-Clin with survival.** **(a)** Event-free survival; **(b)** Overall survival. The size of the squares is proportional to the weight of each dataset.HR,

hazard ratio; 95%-CI, 95% confidence intervals.

Figure S5. **Distribution of immune cell subpopulations derived from CIBERSORTx across IGG groups in the METABRIC, TCGA, GSE58812, and GSE21653 datasets.**

**METABRIC (N=267)**

**TCGA (N=118)**


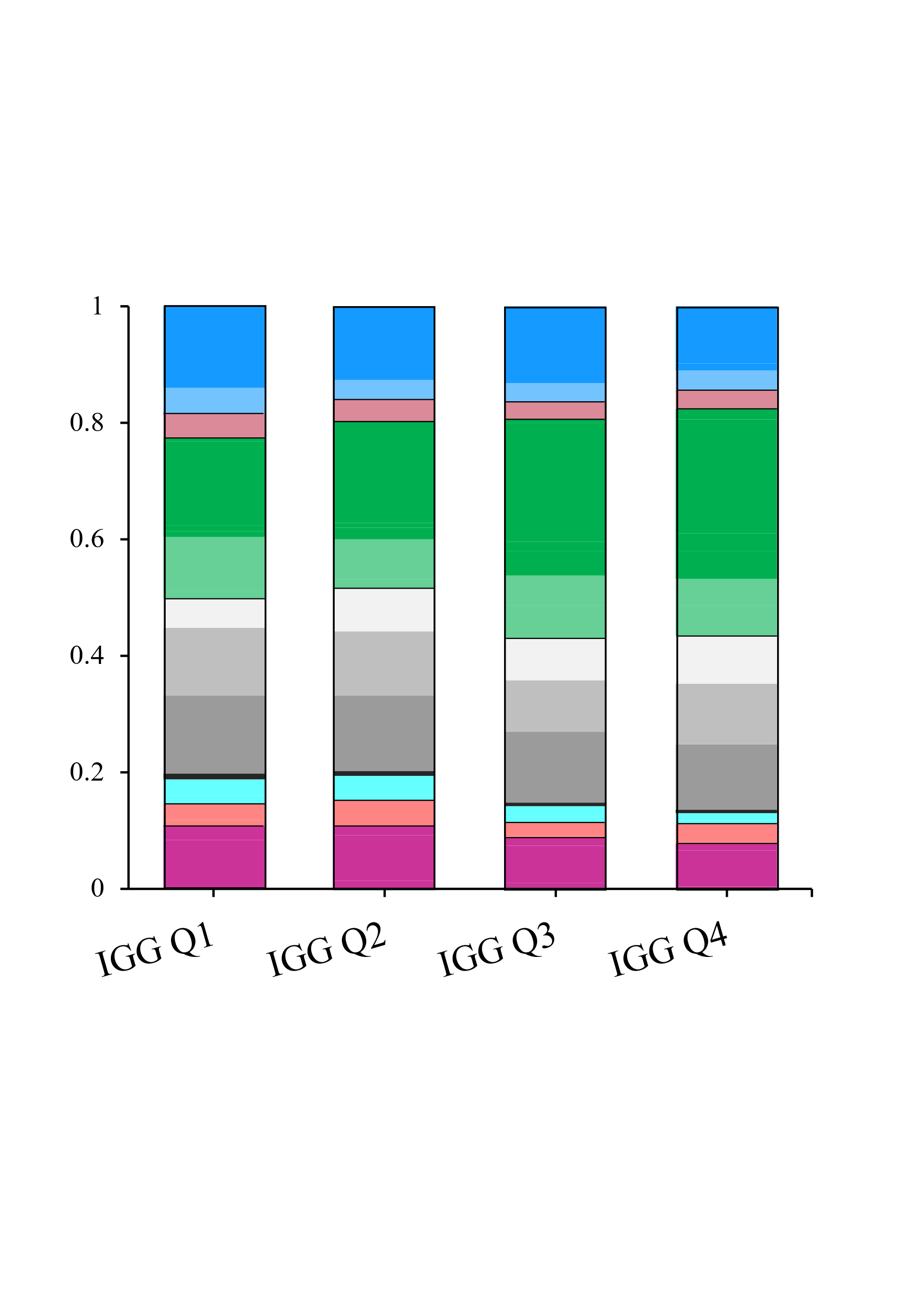

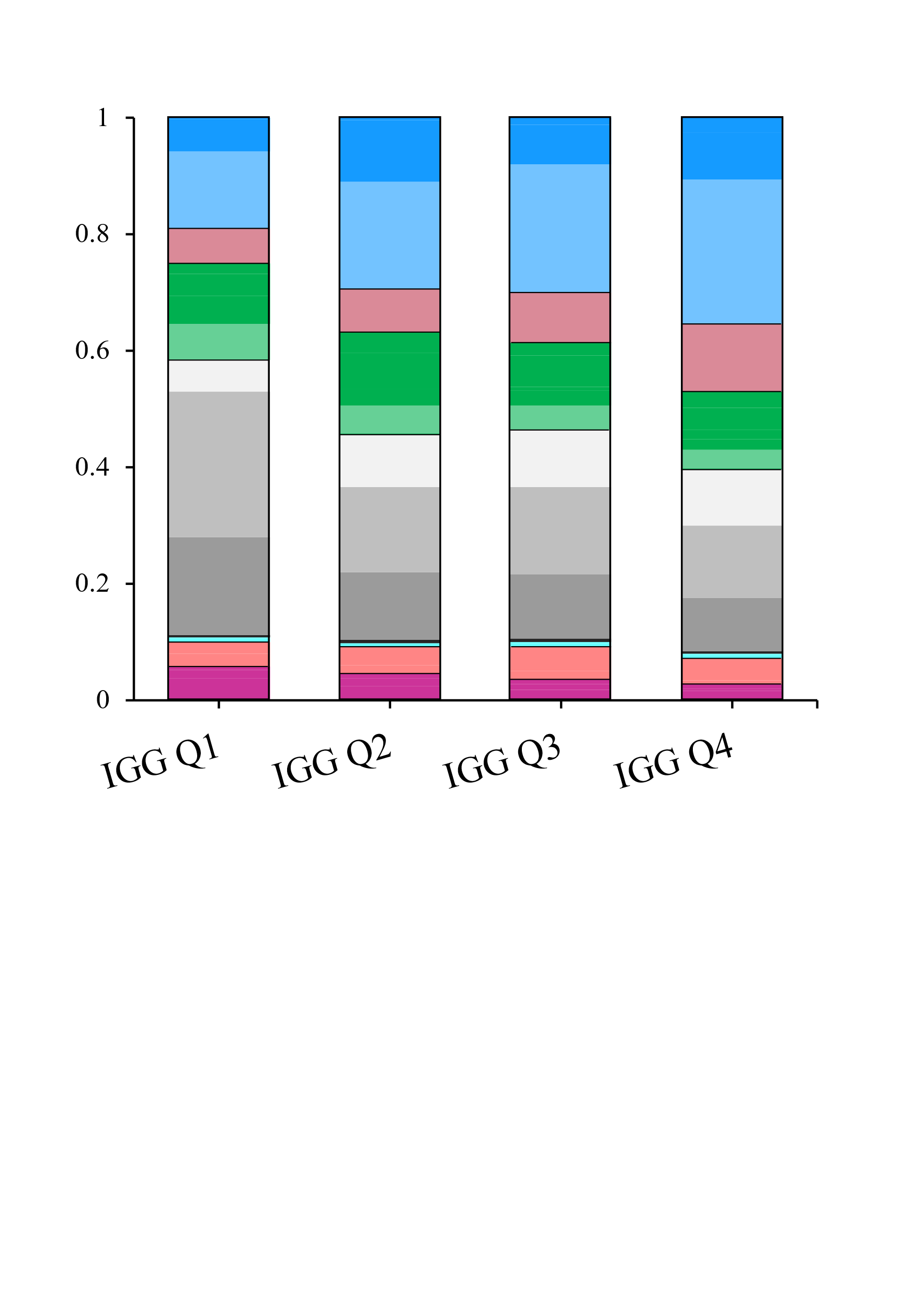


**GSE21653 (N=67)**

**GSE58812 (N=107)**


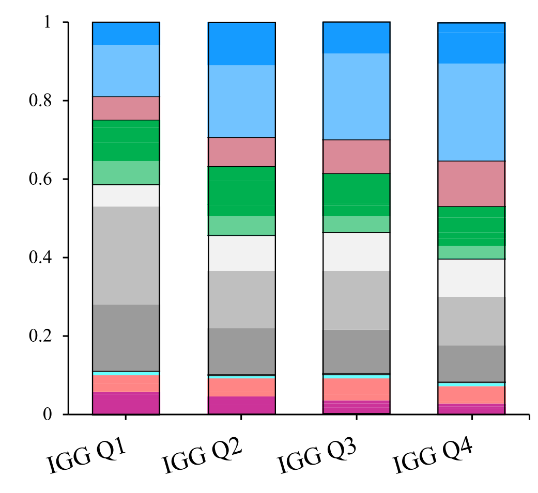


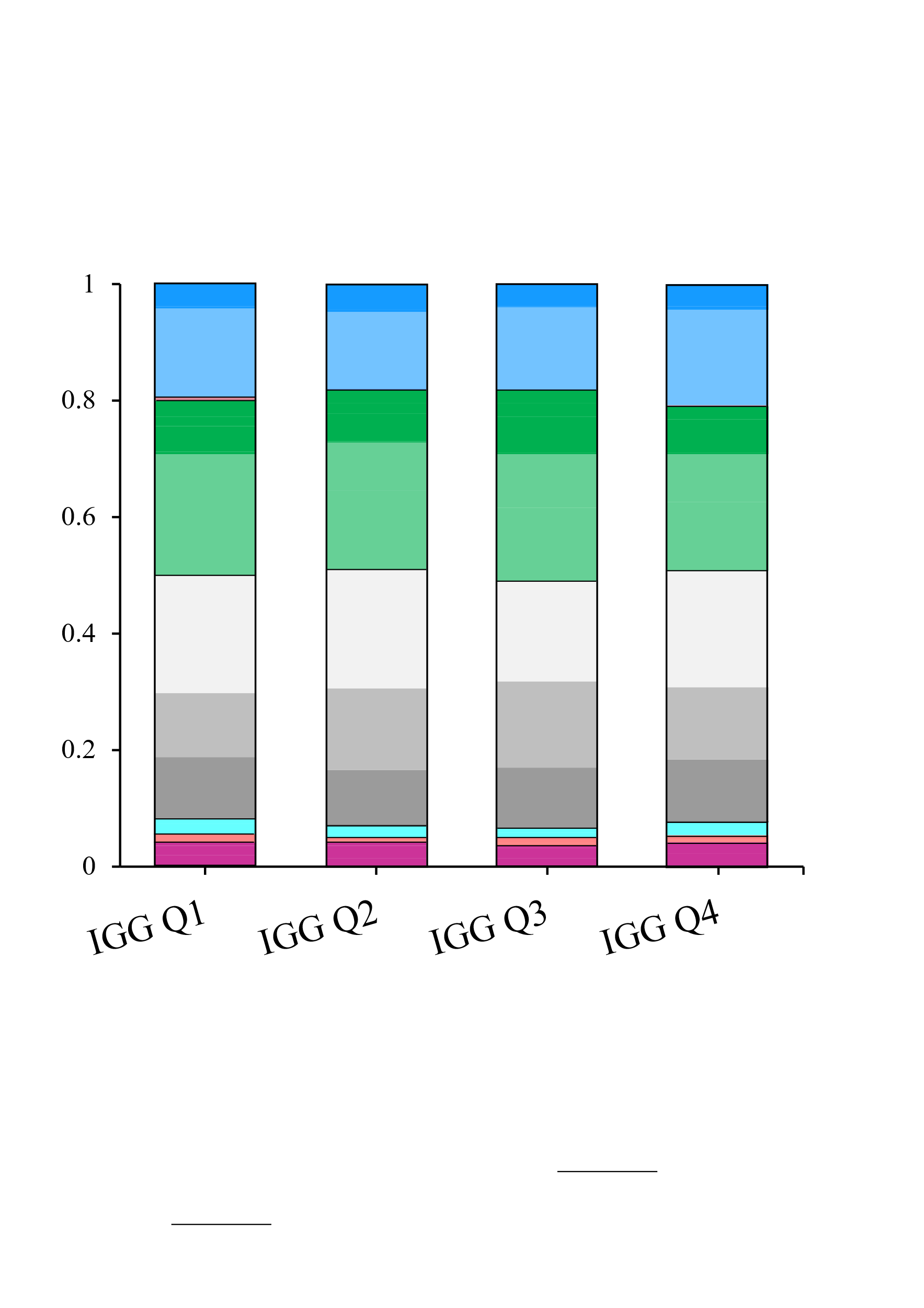


^
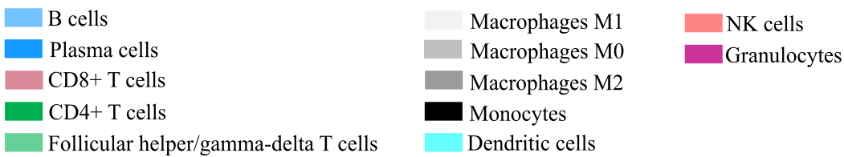
^


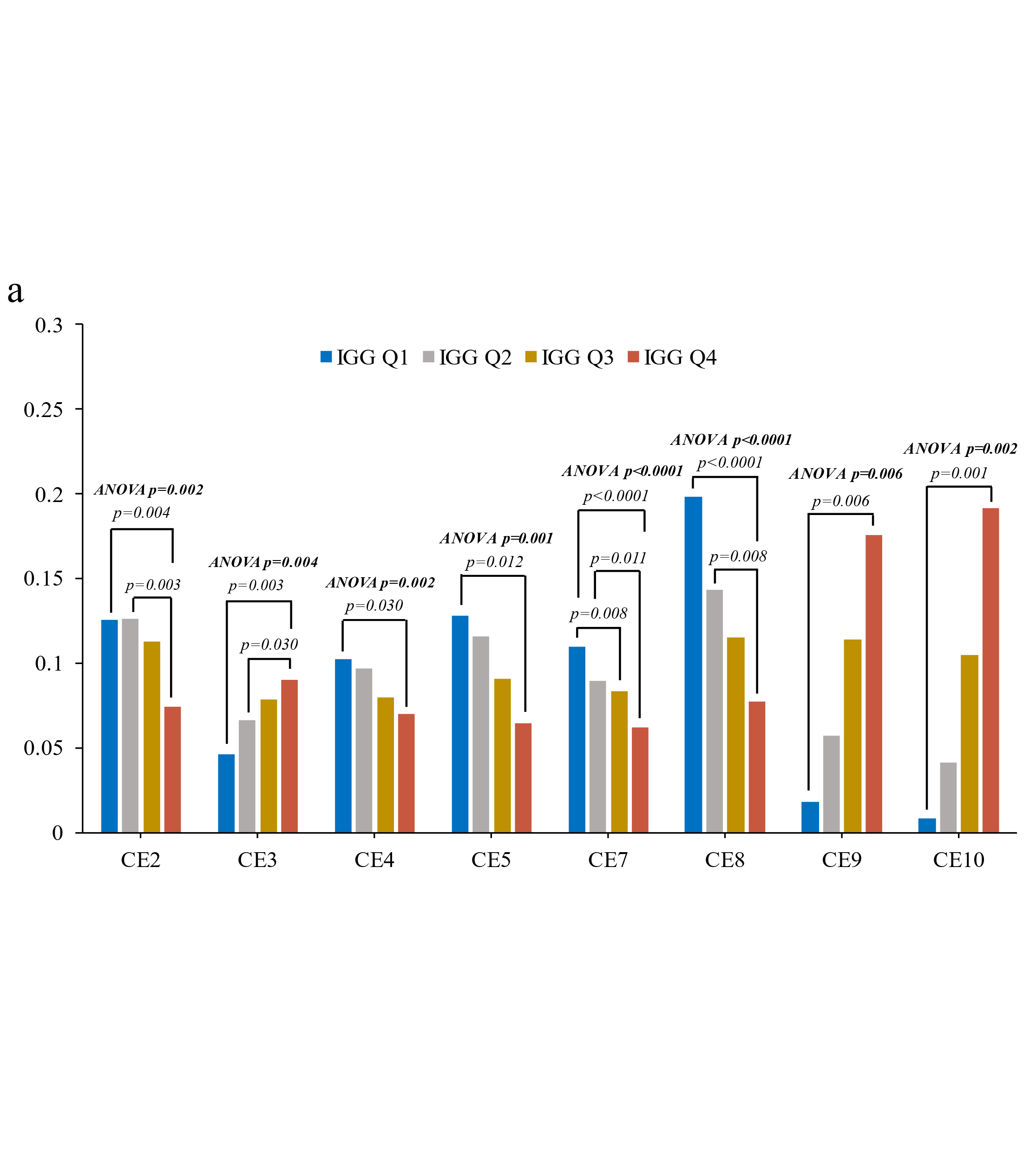
Figure S6. **Distribution of immune ecotypes derived from Ecotyper across IGG groups in the SCAN-B, BrighTNess, CALGB-40603, METABRIC, TCGA, GSE58812, and GSE21653 datasets. a)** Significant changes in immune ecotypes as determined by Ecotyper across IGG quartiles. Statistical differences were evaluated using a 2-way ANOVA considering both IGG quartiles and datasets, followed by Tukey’s honest significant difference analysis (only significant p values showed); **b)** Summary of the distribution of immune ecotypes across IGG quartiles. Percentages were calculated by averaging the expression of each cell type in each IGG quartile, with weighting based on the number of patients in each dataset. Carcinoma ecotype classification spans from extremely immune-cold (1-3) to highly immune-reactive ecotypes (9-10).


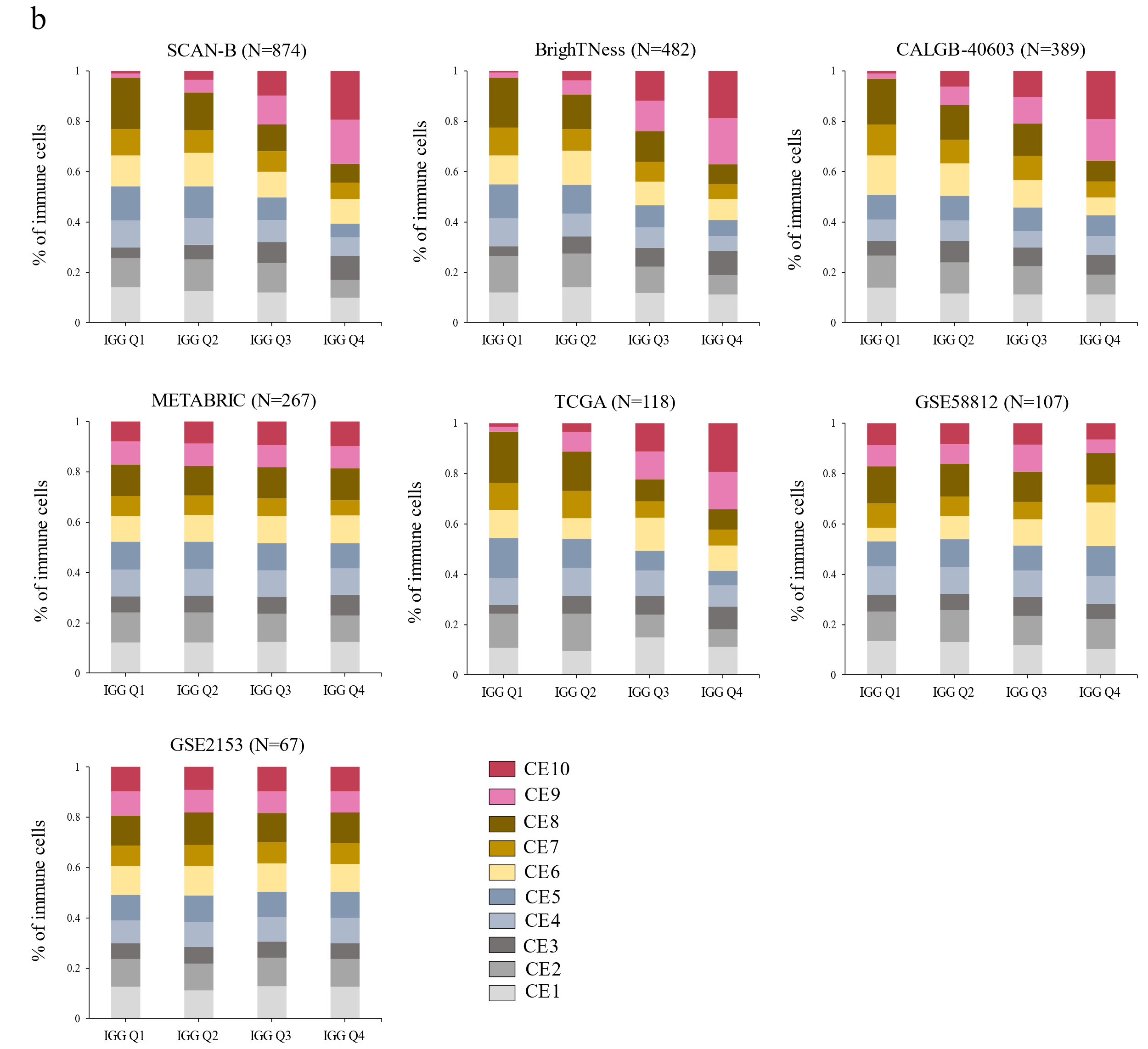


**Figure S7**. **Identification of essential immune genes consistently associated with EFS in SCAN-B and CALGB-40603 and with pCR in CALGB-40603 and BrighTNess.**


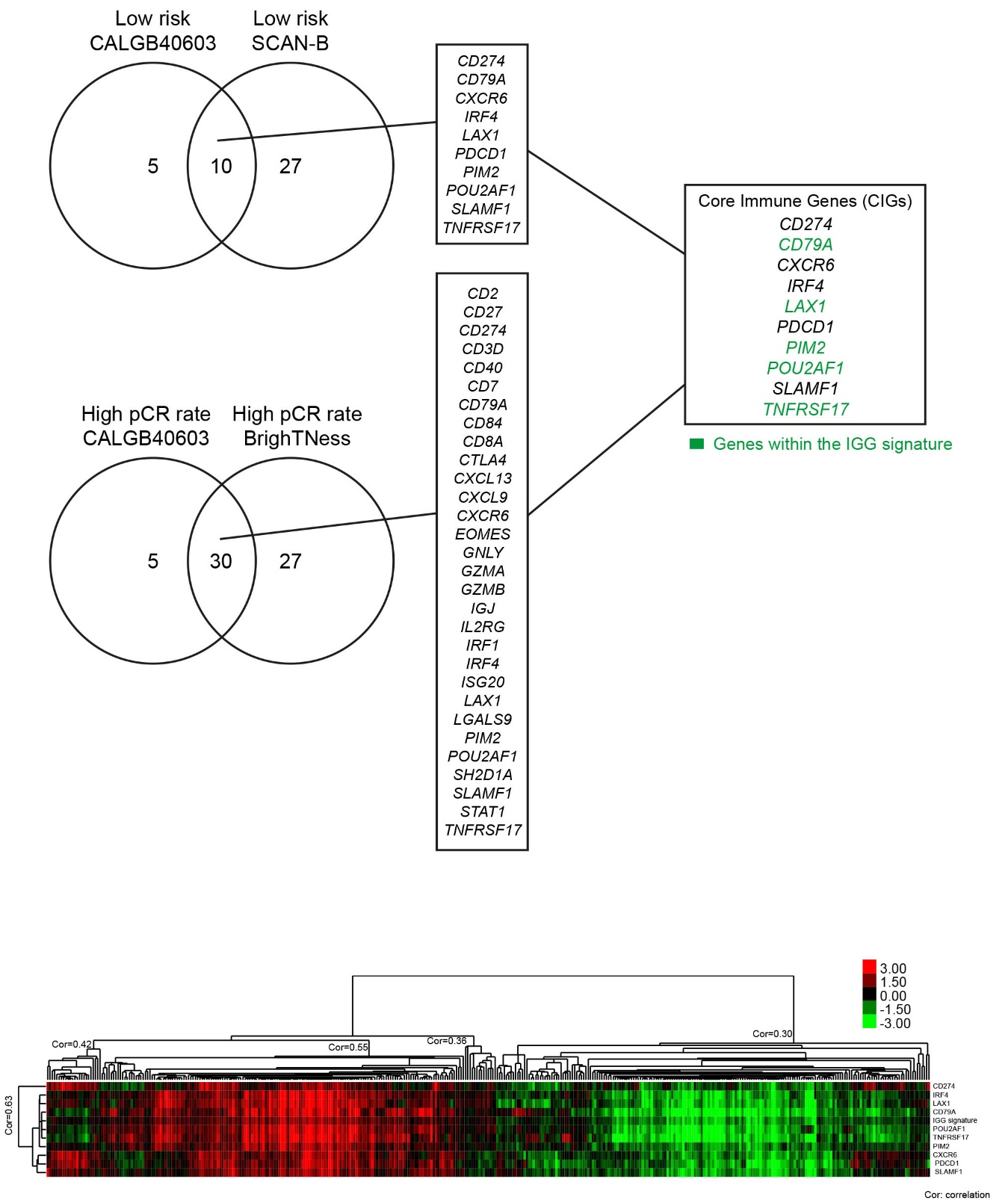


**
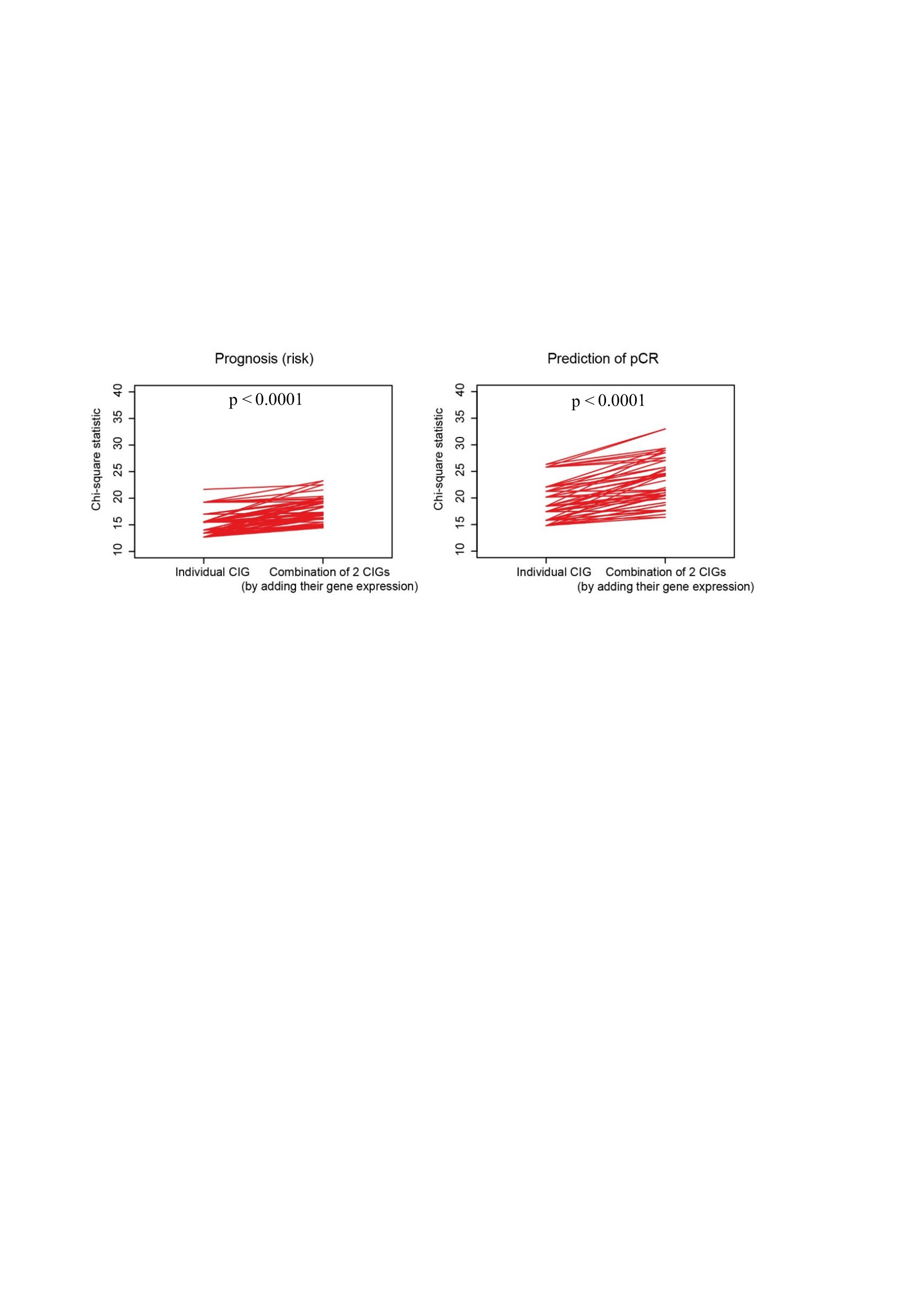
Figure S8. Amount of variation explained in prognosis and prediction of pCR defined by chi-square (χ2) statistics from likelihood ratio test.**

**Table S1. Genomic, response and survival data across datasets.**

| **Source** | **cBioPortal** | | | **GEO omnibus** | | | | | | **dbGAP** |
| --- | --- | --- | --- | --- | --- | --- | --- | --- | --- | --- |
| **Dataset** | **TCGA** | **METABRIC** | | **GSE21653** | **GSE58812** | | **SCAN-B** | | **BrighTNess** | **CALGB-40603** |
| **Accession number** | - | - | | - | - | | - | | GSE164458 | phs001175 |
| **Gene expression platform** | RNA-seq | Microarray (Illumina) | | Microarray  (Affymetrix) | Microarray  (Affymetrix) | | RNA-seq | | RNA-seq | RNA-seq |
| **Gene expression**  **data type** | z-scores transformed expression | | | RMA-averaged, quantile normalized,  Log2-trasformed | MAS 5-0  normalized,  Log2-transformed | | Log-2 transformed | | Log2-normalized abundance data | Salmon-nomalized, Log2-transfromed |
| **ER and PgR cutoff for TNBC** | <10% | | <1% | <1% | <1% | | <1% | | <1% | <10% |
| **pCR** |  | |  |  |  | | |  | X | X |
| **Survival endpoints** |  | |  |  |  | | |  |  |  |
| **DFS** | X | |  | X |  | | |  |  |  |
| **RFI** |  | |  |  |  | | | X |  |  |
| **BCSS** |  | | X |  |  | | |  |  |  |
| **EFS** |  | |  |  |  | | |  |  | X |
| **dDFS** |  | |  |  | X | | |  |  |  |
| **OS** | X | | X |  | | X | | X |  |  |

ER, estrogen receptor; PgR, progesterone receptor; DFS, disease-free survival; RFI, relapse-free interval; BCSS, breast cancer specific survival; EFS, event-free survival; dDFS, distant disease-free survvial; OS, overall survival

**Table S2. Number of events, censoring and number at risk for each of the cohorts at the 5-year follow-up mark for both EFS and OS.**

|  | | **5-year Event free survival** | | | **5-year Overall survival** | | |
| --- | --- | --- | --- | --- | --- | --- | --- |
| **Dataset** | **Total N** | **N. events (%)** | **N. censored (%)** | **N at risk (%)** | **N. events (%)** | **N. censored (%)** | **N at risk (%)** |
| **SCAN-B** | 874 | 123 (14) | 378 (43) | 373 (43) | 188 (21) | 138 (16) | 548 (63) |
| **CALGB-40603** | 389 | 107 (28) | 64 (16) | 218 (56) | NA | NA | NA |
| **METABRIC** | 267 | 92 (34) | 15 (6) | 160 (60) | 88 (33) | 7 (3) | 174 (64) |
| **TCGA** | 118 | 19 (16) | 77 (65) | 22 (19) | 15 (13) | 78 (66) | 25 (21) |
| **GSE58812** | 107 | 29 (27) | 19 (18) | 59 (55) | 28 (26) | 19 (18) | 60 (56) |
| **GSE21653** | 87 | 26 (30) | 35 (40) | 26 (30) | NA | NA | NA |

Table S3. **Univariate cox analyses for the relationship between IGG-Clin and event-free survival and overall survival.**

| **Dataset** | **Event-free survival** | | **Overall survival** | |
| --- | --- | --- | --- | --- |
|  | **HR (95% CIs)** | **p value** | **HR (95% CIs)** | **p value** |
| **TCGA** | 2.58 (1.37-4.85) | 0.003 | 3.26 (1.67-6.78) | 0.002 |
| **METABRIC** | 1.99 (1.52-2.62) | <0.0001 | 1.47 (1.17-1.84) | 0.002 |
| **GSE21653** | 3.34 (1.82-6.11) | 0.0001 | NA | NA |
| **GSE58812** | NA | NA | NA | NA |
| **SCAN-B** | 2.17 (1.73-2.72)- | <0.0001 | 2.17 (1.85-2.56) | <0.0001 |
| **CALGB-40603** | 1.86 (1.39-2.49) | <0.0001 | NA | NA |

HR, hazard ratio; CI, confidence intervals; NA, not available.

Table S4. **Characteristics of the CALGB-40603 and BrighTNess datasets. Event free survival events are reported according to the main trial definition.**

|  | **CALGB-40603** | **BrighTNess** |
| --- | --- | --- |
| **N** | 389 | 482 |
| **Tumor size, N (%)** |  |  |
| T1 | 42 (11) | - |
| T2-4 | 340 (87) | - |
| NA | 7 (2) | 482 (100) |
| **Nodal status, N (%)** |  |  |
| N0 | 164 (42) | 288 (51) |
| N+ | 201 (52) | 194 (49) |
| NA | 24 (6) | 0 (0) |
| **Neoadjuvant carboplatin, N (%)** |  |  |
| Yes | 201 (52) | 359 (74) |
| No | 188 (48) | 123 (26) |
| NA | 0 (0) | 0 (0) |
| **pCR, N (%)** | 186 (48) | 236 (49) |
| **Residual disease**  RCB-I  RCB-II  RCB-III  NA | 203 (52) | 246 (51) |
|  | 49 (24) | 46 (19) |
|  | 100 (49) | 124 (50) |
|  | 31 (15) | 20 (8) |
|  | 23 (12) | 56 (23) |
| **Median follow-up**, **years** | 5·7 (4·8-6·4) | NA |
| **EFSl events** | 115 | NA |

NA, not available; pCR, pathologic complete response; RCB, residual cancer burden; EFS, event free survival.

**Table S5. pCR rates according to IGG signature expression in the CALGB-40603 and BrighTNess datasets.**

| **Dataset** | **IGG/immune expression quartiles** | | | | **Chi-square test,**  **p value** |
| --- | --- | --- | --- | --- | --- |
|  | **Q1 -Ultralow** | **Q2-Low** | **Q3-High** | **Q4-Ultrahigh** |  |
| **CALGB-40603, N=389**  **pCR, N (%)** | 37 (37.8) | 41 (42.3) | 49 (50.5) | 59 (60.8) | 0.007 |
| **BrighTNess, N=482**  **pCR, N= (%)** | 44 (36.0) | 58 (48.0) | 62 (52.0) | 72 (60.0) | 0.003 |

pCR, pathologic complete response

**Table S6. Distribution of immune cells as results of a 2-way ANOVA considering both IGG quartiles and datasets, followed by post-hoc Tukey’s test.**

| **Population** | **ANOVA  p value** | **TUKEY'S TEST, p values** | | | | | |
| --- | --- | --- | --- | --- | --- | --- | --- |
|  |  | **Q1Q2** | **Q1Q3** | **Q1Q4** | **Q2Q3** | **Q2Q4** | **Q3Q4** |
| B.cells.memory | 0.169 | 0.880 | 0.402 | 0.159 | 0.776 | 0.375 | 0.854 |
| B.cells.naive | 0.904 | 0.999 | 0.973 | 0.960 | 0.944 | 0.924 | 1.000 |
| Dendritic.cells.activated | 0.080 | 0.980 | 0.723 | 0.080 | 0.899 | 0.125 | 0.284 |
| Dendritic.cells.resting | 0.514 | 0.994 | 0.926 | 0.640 | 0.829 | 0.513 | 0.926 |
| Eosinophils | 0.304 | 0.595 | 0.344 | 0.337 | 0.947 | 0.942 | 1.000 |
| Macrophages.M0 | 0.004 | 0.095 | 0.045 | 0.044 | 0.274 | 0.067 | 0.661 |
| Macrophages.M1 | 0.035 | 0.249 | 0.010 | 0.003 | 0.513 | 0.519 | 1.000 |
| Macrophages.M2 | 0.003 | 0.065 | 0.011 | 0.003 | 0.418 | 0.070 | 0.482 |
| Mast.cells.activated | 0.017 | 0.676 | 0.085 | 0.017 | 0.336 | 0.061 | 0.522 |
| Mast.cells.resting | 0.058 | 0.809 | 0.108 | 0.080 | 0.315 | 0.233 | 0.993 |
| Monocytes | 0.163 | 0.920 | 0.504 | 0.149 | 0.829 | 0.313 | 0.720 |
| Neutrophils | 0.219 | 0.599 | 0.388 | 0.189 | 0.970 | 0.727 | 0.922 |
| NK.cells.activated | 0.468 | 0.860 | 0.565 | 0.469 | 0.937 | 0.868 | 0.997 |
| NK.cells.resting | 0.835 | 0.913 | 0.901 | 1.000 | 1.000 | 0.920 | 0.909 |
| Plasma.cells | 0.004 | 0.545 | 0.113 | 0.004 | 0.553 | 0.012 | 0.058 |
| T.cells.CD4.memory.activated | 0.754 | 0.999 | 1.000 | 0.781 | 0.999 | 0.850 | 0.793 |
| T.cells.CD4.memory.resting | 0.740 | 0.813 | 0.763 | 0.984 | 1.000 | 0.948 | 0.917 |
| T.cells.CD4.naive | 0.771 | 1.000 | 0.833 | 0.931 | 0.817 | 0.920 | 0.994 |
| T.cells.CD8 | 0.002 | 0.115 | 0.009 | 0.001 | 0.215 | 0.053 | 0.014 |
| T.cells.follicular.helper | 0.040 | 0.416 | 0.045 | 0.060 | 0.320 | 0.427 | 0.993 |
| T.cells.gamma.delta | 0.353 | 0.994 | 0.765 | 0.357 | 0.880 | 0.465 | 0.843 |
| T.cells.regulatory..Tregs. | 0.371 | 0.966 | 0.739 | 0.344 | 0.935 | 0.552 | 0.850 |

**Table S7. Distribution of Ecotypes as results of a 2-way ANOVA considering both IGG quartiles and datasets, followed by post-hoc Tukey’s test.**

| **Ecotypes** | **ANOVA p value** | **TUKEY'S TEST, p values** | | | | | |
| --- | --- | --- | --- | --- | --- | --- | --- |
|  |  | **Q1Q2** | **Q1Q3** | **Q1Q4** | **Q2Q3** | **Q2Q4** | **Q3Q4** |
| CE1 | 0.274 | 0.798 | 0.975 | 0.246 | 0.959 | 0.734 | 0.443 |
| CE2 | 0.002 | 0.996 | 0.346 | 0.004 | 0.252 | 0.003 | 0.140 |
| CE3 | 0.004 | 0.117 | 0.053 | 0.003 | 0.861 | 0.030 | 0.722 |
| CE4 | 0.002 | 0.972 | 0.153 | 0.030 | 0.307 | 0.064 | 0.805 |
| CE5 | 0.001 | 0.854 | 0.124 | 0.012 | 0.430 | 0.077 | 0.718 |
| CE6 | 0.966 | 0.998 | 0.997 | 0.989 | 0.983 | 0.964 | 0.999 |
| CE7 | 0.000 | 0.192 | 0.008 | 0.000 | 0.387 | 0.011 | 0.246 |
| CE8 | 0.000 | 0.158 | 0.069 | 0.000 | 0.461 | 0.008 | 0.655 |
| CE9 | 0.006 | 0.640 | 0.069 | 0.006 | 0.476 | 0.069 | 0.640 |
| CE10 | 0.002 | 0.871 | 0.100 | 0.001 | 0.349 | 0.052 | 0.248 |

**Table S8. List of interrogated genes from the 185-genes panel.**

| **Gene list** | **Interrogated in CALGB40603** | **Interrogated in SCAN-B** | **Interrogated in BrighTNess** |
| --- | --- | --- | --- |
| *ABCC11* | yes | yes | yes |
| *ACTG2* | yes | yes | yes |
| *ACTR3B* | yes | yes | yes |
| *AFF3* | yes | yes | yes |
| *AGR2* | yes | yes | yes |
| *AGR3* | yes | yes | yes |
| *ANLN* | yes | yes | yes |
| *AR* | yes | yes | yes |
| *ASPM* | yes | yes | yes |
| *AURKA* | yes | yes | yes |
| *BAG1* | yes | yes | yes |
| *BCL2* | yes | yes | yes |
| *BIRC5* | yes | yes | yes |
| *BLVRA* | yes | yes | yes |
| *BOC* | yes | yes | yes |
| *BRCA1* | yes | yes | yes |
| *BRCA2* | yes | yes | yes |
| *BUB1* | yes | yes | yes |
| *C2orf54* | yes | yes | yes |
| *CCNB1* | yes | yes | yes |
| *CCNB2* | yes | yes | yes |
| *CCND1* | yes | yes | yes |
| *CCNE1* | yes | yes | yes |
| *CD19* | yes | yes | yes |
| *CD2* | yes | yes | yes |
| *CD27* | yes | yes | yes |
| *CD274* | yes | yes | yes |
| *CD3D* | yes | yes | yes |
| *CD3G* | yes | yes | yes |
| *CD4* | yes | yes | yes |
| *CD40* | yes | yes | yes |
| *CD68* | yes | yes | no |
| *CD7* | yes | yes | yes |
| *CD79A* | yes | yes | yes |
| *CD84* | yes | yes | yes |
| *CD86* | yes | yes | yes |
| *CD8A* | yes | yes | yes |
| *CDC20* | yes | yes | yes |
| *CDC6* | yes | yes | yes |
| *CDCA1* | yes | yes | yes |
| *CDCA5* | yes | yes | yes |
| *CDCA8* | yes | yes | yes |
| *CDH3* | yes | yes | yes |
| *CDKN3* | yes | yes | yes |
| *CENPA* | yes | yes | yes |
| *CENPF* | yes | yes | yes |
| *CEP55* | yes | yes | yes |
| *CLUAP1* | yes | yes | yes |
| *CNTNAP2* | yes | yes | no |
| *CREB3L4* | yes | yes | yes |
| *CRYAB* | yes | yes | yes |
| *CTLA4* | yes | yes | yes |
| *CX3CL1* | yes | yes | yes |
| *CXCL13* | yes | yes | yes |
| *CXCL8* | yes | yes | yes |
| *CXCL9* | yes | yes | yes |
| *CXCR6* | yes | yes | yes |
| *CXXC5* | yes | yes | yes |
| *DGKD* | yes | yes | yes |
| *DNAJC12* | yes | yes | yes |
| *DNALI1* | yes | yes | yes |
| *E2F1* | yes | yes | yes |
| *EAF2* | yes | yes | yes |
| *EGFR* | yes | yes | yes |
| *EOMES* | yes | yes | yes |
| *ERBB2* | yes | yes | yes |
| *ERBB3* | yes | yes | yes |
| *ERBB4* | yes | yes | yes |
| *ESR1* | yes | yes | yes |
| *ETFA* | yes | yes | yes |
| *EXO1* | yes | yes | yes |
| *F12* | yes | yes | yes |
| *FA2H* | yes | yes | yes |
| *FGFR1* | yes | yes | yes |
| *FGFR2* | yes | yes | yes |
| *FGFR4* | yes | yes | yes |
| *FHOD1* | yes | yes | yes |
| *FOXA1* | yes | yes | yes |
| *FOXC1* | yes | yes | yes |
| *GABRP* | yes | yes | yes |
| *GARS* | yes | yes | yes |
| *GATA3* | yes | yes | yes |
| *GNLY* | yes | yes | yes |
| *GPNMB* | yes | yes | yes |
| *GPR160* | yes | yes | yes |
| *GRB7* | yes | yes | yes |
| *GSDMB* | yes | yes | yes |
| *GZMA* | yes | yes | yes |
| *GZMB* | yes | yes | yes |
| *HLA.C* | yes | yes | yes |
| *ID4* | yes | yes | yes |
| *IGJ* | yes | yes | yes |
| *IGKC* | yes | no | no |
| *IGL* | no | no | no |
| *IGLV3.25* | yes | no | no |
| *IL18R1* | yes | yes | yes |
| *IL23A* | yes | yes | yes |
| *IL2RG* | yes | yes | yes |
| *IL34* | yes | yes | yes |
| *IRF1* | yes | yes | yes |
| *IRF4* | yes | yes | yes |
| *IRF8* | yes | yes | yes |
| *ISG20* | yes | yes | yes |
| *ITK* | yes | yes | yes |
| *KCTD9* | yes | yes | yes |
| *KIF23* | yes | yes | yes |
| *KIF2C* | yes | yes | yes |
| *KLK5* | yes | yes | yes |
| *KLRB1* | yes | yes | yes |
| *KLRD1* | yes | yes | no |
| *KNTC2* | yes | yes | yes |
| *KRT14* | yes | yes | yes |
| *KRT17* | yes | yes | yes |
| *KRT18* | yes | yes | yes |
| *KRT5* | yes | yes | yes |
| *KRT6B* | yes | yes | yes |
| *KYNU* | yes | yes | yes |
| *LAX1* | yes | yes | yes |
| *LGALS9* | yes | yes | yes |
| *LY9* | yes | yes | yes |
| *MAGED2* | yes | yes | yes |
| *MAPT* | yes | yes | no |
| *MDM2* | yes | yes | yes |
| *MELK* | yes | yes | yes |
| *MFSD2A* | yes | yes | yes |
| *MIA* | yes | yes | yes |
| *MID1* | yes | yes | yes |
| *MKI67* | yes | yes | yes |
| *MLPH* | yes | yes | yes |
| *MMP1* | yes | yes | yes |
| *MMP11* | yes | yes | yes |
| *MND1* | yes | no | yes |
| *MPHOSPH6* | yes | yes | yes |
| *MRAS* | yes | yes | yes |
| *MSLN* | yes | yes | yes |
| *MUCL1* | yes | yes | yes |
| *MYBL2* | yes | yes | yes |
| *MYC* | yes | yes | yes |
| *NAT1* | yes | yes | yes |
| *NDRG2* | yes | yes | yes |
| *NECTIN4* | no | no | yes |
| *NEK2* | yes | yes | yes |
| *NFIB* | yes | yes | yes |
| *NQO1* | yes | yes | yes |
| *NTN3* | yes | yes | yes |
| *ORC6L* | yes | yes | yes |
| *ORMDL3* | yes | yes | yes |
| *PDCD1* | yes | yes | no |
| *PGR* | yes | yes | yes |
| *PHGDH* | yes | yes | yes |
| *PIM2* | yes | yes | yes |
| *PNMT* | yes | yes | yes |
| *POU2AF1* | yes | yes | yes |
| *PSMD3* | yes | yes | yes |
| *PTTG1* | yes | yes | yes |
| *RAD51* | yes | yes | yes |
| *RB1* | yes | yes | yes |
| *RRAGA* | yes | yes | yes |
| *RRM2* | yes | yes | yes |
| *S100A9* | yes | yes | yes |
| *SERPINB5* | yes | yes | yes |
| *SFRP1* | yes | yes | yes |
| *SH2D1A* | yes | yes | yes |
| *SIAH2* | yes | yes | yes |
| *SLAMF1* | yes | yes | yes |
| *SLC39A6* | yes | yes | yes |
| *SPDEF* | yes | yes | yes |
| *STARD3* | yes | yes | yes |
| *STAT1* | yes | yes | yes |
| *STAT4* | yes | yes | yes |
| *TCAP* | yes | yes | yes |
| *TFCP2L1* | yes | yes | yes |
| *THSD4* | yes | yes | yes |
| *TMEM45B* | yes | yes | yes |
| *TNFRSF17* | yes | yes | yes |
| *TOP2A* | yes | yes | yes |
| *TROP2* | no | yes | yes |
| *TRPV6* | yes | yes | yes |
| *TSPAN13* | yes | yes | yes |
| *TTK* | yes | yes | yes |
| *TYMS* | yes | yes | yes |
| *UBE2C* | yes | yes | yes |
| *UBE2T* | yes | yes | yes |
| *XBP1* | yes | yes | yes |
| *ZNF552* | yes | yes | yes |

Table S9. **Amount of variation explained in prognosis defined by chi-square (χ2) statistics from likelihood ratio tests.**

| **Combination of 2 CIGs** | **Gene 1** | **Gene 2** | **Sum** | **Substract** | **Multiply** | **Ratio** |
| --- | --- | --- | --- | --- | --- | --- |
| *CD79A_IRF4* | 15.81 | 21.24 | 19.14 | 0.39 | 0.17 | 1.16 |
| *CD79A_CD274* | 15.81 | 25.80 | 25.49 | 1.13 | 0.06 | 0.30 |
| *CD79A_CXCR6* | 15.81 | 22.08 | 21.95 | 1.53 | 1.44 | 0.42 |
| *CD79A_LAX1* | 15.81 | 20.17 | 19.14 | 0.01 | 0.21 | 1.49 |
| *CD79A_PIM2* | 15.81 | 26.32 | 21.02 | 1.52 | 0.04 | 2.99 |
| *CD79A_POU2AF1* | 15.81 | 18.44 | 17.56 | 3.79 | 0.20 | 3.03 |
| *CD79A_SLAMF1* | 15.81 | 17.43 | 17.70 | 5.89 | 0.06 | 4.86 |
| *CD79A_TNFRSF17* | 15.81 | 14.80 | 16.39 | 0.51 | 1.24 | 0.37 |
| *IRF4_CD274* | 21.24 | 25.80 | 28.52 | 1.04 | 0.21 | 0.06 |
| *IRF4_CXCR6* | 21.24 | 22.08 | 25.78 | 1.23 | 2.34 | 0.30 |
| *IRF4_LAX1* | 21.24 | 20.17 | 21.38 | 0.57 | 0.60 | 1.72 |
| *IRF4_PIM2* | 21.24 | 26.32 | 24.64 | 1.25 | 0.10 | 4.48 |
| *IRF4_POU2AF1* | 21.24 | 18.44 | 20.97 | 2.37 | 0.66 | 0.84 |
| *IRF4_SLAMF1* | 21.24 | 17.43 | 21.45 | 5.87 | 0.31 | 1.01 |
| *IRF4_TNFRSF17* | 21.24 | 14.80 | 18.70 | 1.23 | 2.57 | 0.47 |
| *CD274_CXCR6* | 25.80 | 22.08 | 27.59 | 0.00 | 2.36 | 0.10 |
| *CD274_LAX1* | 25.80 | 20.17 | 27.04 | 1.76 | 0.34 | 0.01 |
| *CD274_PIM2* | 25.80 | 26.32 | 32.98 | 0.20 | 0.00 | 1.27 |
| *CD274_POU2AF1* | 25.80 | 18.44 | 28.97 | 0.02 | 0.65 | 2.43 |
| *CD274_SLAMF1* | 25.80 | 17.43 | 25.19 | 0.97 | 0.28 | 2.62 |
| *CD274_TNFRSF17* | 25.80 | 14.80 | 24.49 | 1.72 | 3.08 | 0.25 |
| *CXCR6_LAX1* | 22.08 | 20.17 | 24.32 | 2.21 | 2.29 | 0.56 |
| *CXCR6_PIM2* | 22.08 | 26.32 | 29.34 | 0.24 | 1.22 | 1.13 |
| *CXCR6_POU2AF1* | 22.08 | 18.44 | 24.39 | 0.04 | 3.28 | 3.18 |
| *CXCR6_SLAMF1* | 22.08 | 17.43 | 21.62 | 1.48 | 2.58 | 1.39 |
| *CXCR6_TNFRSF17* | 22.08 | 14.80 | 20.63 | 2.44 | 5.48 | 0.00 |
| *LAX1_PIM2* | 20.17 | 26.32 | 24.13 | 2.09 | 0.21 | 5.12 |
| *LAX1_POU2AF1* | 20.17 | 18.44 | 20.52 | 3.77 | 0.79 | 0.65 |
| *LAX1_SLAMF1* | 20.17 | 17.43 | 20.52 | 8.03 | 0.36 | 2.89 |
| *LAX1_TNFRSF17* | 20.17 | 14.80 | 18.67 | 0.50 | 2.54 | 0.12 |
| *PIM2_POU2AF1* | 26.32 | 18.44 | 23.29 | 0.19 | 0.31 | 1.49 |
| *PIM2_SLAMF1* | 26.32 | 17.43 | 24.51 | 2.87 | 0.06 | 0.86 |
| *PIM2_TNFRSF17* | 26.32 | 14.80 | 20.42 | 2.24 | 1.98 | 0.14 |
| *POU2AF1_SLAMF1* | 18.44 | 17.43 | 19.90 | 1.70 | 0.57 | 1.84 |
| *POU2AF1_TNFRSF17* | 18.44 | 14.80 | 16.95 | 4.79 | 2.54 | 0.12 |
| *SLAMF1_TNFRSF17* | 17.43 | 14.80 | 17.59 | 5.45 | 2.15 | 0.29 |
